# An enhanced broadband class-J mode power amplifier for 5G smart meter applications

**DOI:** 10.12688/f1000research.73498.2

**Published:** 2024-09-12

**Authors:** Nagisetty Sridhar, Dr Chinnaiyan Senthilpari, Dr Mardeni R, Dr Wong Hin Yong

**Affiliations:** 1 FACULTY OF ENGINEERING, MULTIMEDIA UNIVERSITY, CYBERJAYA, SELANGOR, 63100, Malaysia

**Keywords:** 5G, 3.5 GHz, power amplifier, good efficiency, wide bandwidth, Class-J, matching networks

## Abstract

**Background:** With the tremendous increase in the usage of smart meters for industrial/ household purposes, their implementation is considered a crucial challenge in the Internet of Things (IoT) world, leading to a demand for emerging 5G technology. In addition, a large amount of data has to be communicated by smart meters efficiently, which needs a significant enhancement in bandwidth. The power amplifier (PA) plays a major role in deciding the efficiency and bandwidth of the entire communication system. Among the various modes of PAs, a newly developed Class-J mode PA has been proven to achieve high efficiency over a wide bandwidth by maintaining linearity.

**Methods:** This paper proposes a Class-J mode PA design methodology using a CGH40010F-GaN device that operates at a 3.5 GHz frequency to meet the requirements of 5G wireless communication technology for the replacement of existing 4G/LTE technology used for advanced metering infrastructure (AMI) in smart grids. This research's main objective is to design the proper matching networks (M.Ns) to achieve Class-J mode operation that satisfies the bandwidth requirements of 5G smart grid applications. With the target impedances obtained using the load-pull simulation, lumped element matching networks are analyzed and designed in 3 ways using the ADS EDA tool.

**Results:** The simulation results reveal that the proposed Class-J PA provides a maximum drain efficiency (D.E) of 82%, power added efficiency (PAE) of 67% with 13 dB small-signal gain at 3.5 GHz, and output power of 40 dBm (41.4 dBm peak) with a power gain of approximately 7 dB over a bandwidth of approximately 400 MHz with a 28 V power supply into a 50 Ω load.

**Conclusion:** The efficiency and bandwidth of the proposed Class-J PA can be enhanced further by fine-tuning the matching network design to make it more suitable for 5G smart meter/grid applications.

## Introduction

With the tremendous improvement in the wireless communication industry, the demand for emerging 5G technology ^1^^–^^3^ has increased for enhanced broadband and Internet of Things (IoT) applications. Today, many smart devices introduce intelligent behavior into households and industrial equipment to realize smart grids and smart homes/cities. For example, a smart energy meter can measure energy consumption by one device and send the data to the energy provider along with the end consumer statistics using two-way wireless communication technology. Although the existing 4G technology has served us well to date, it cannot satisfy the new challenges that can be brought by emerging wireless communication applications such as 5G smart grids, as a large amount of data transfer is needed at higher data rates with low latency. Hence, the adoption of 5G technology becomes more crucial. Therefore, worldwide research has started to implement this 5G technology by adopting techniques such as millimeter waves, small cells, massive MIMO, beamforming, and full duplexing. ^4^ However, the two major challenges to be solved while implementing this 5G technology for these applications are a reduction in energy consumption ^5^ and an enhancement in bandwidth. As the PAE of the power amplifiers ^6^^,^^7^ used in the R.F. transmitter plays the key role in deciding the energy consumption by the overall 5G wireless communication network, a power amplifier is needed that can provide improvement in PAE without compromising linearity and bandwidth. Many PA topologies have been reported previously ^8^^–^^14^ with various techniques to achieve linear amplification with high efficiency. Switching mode PAs such as Class-E/F demonstrate potential in providing excellent PAE but are not beneficial for enhanced bandwidth 5G applications because of their narrow bandwidth nature of harmonic terminations. On the other hand, linear mode PAs such as Class-A/B can achieve lower efficiency but are more linear than switching mode PAs. Nevertheless, Class-B mode PAs with harmonic tuning can theoretically achieve a peak efficiency of approximately 78.5%, and their bandwidth is also limited, as harmonic termination is difficult to realize over a wide bandwidth. However, this study mainly focuses on the design of a power amplifier that satisfies the requirements of smart grid wireless communication networks that ^15^^,^^16^ used for AMI of smart meters. A distributed communication architecture that can provide cost-effective and efficient communication is proposed in. ^17^ Distributed operation centers presently use 3G/4G cellular networks to communicate with data concentrators, which can be replaced by emerging 5G communication technology to support the increase in data traffic. A comprehensive review of 5G wireless communication networks for smart grids is presented in, ^18^ which motivates PA designers to design a PA that overcomes the bandwidth limitations of existing linear and switching mode PAs and satisfies the bandwidth requirements of 5G wireless communications in smart grids. A newly developed Class-J mode PA by S.C. Cripps in ^19^ has proven its potential to achieve high efficiency without compromising linearity and bandwidth. The design methodology for highly efficient, linear, and broadband Class-J PA mode is demonstrated in. ^20^ Different Class-J mode PAs were reviewed to analyze their suitability for near-future 5G wireless communications used for smart grid applications. Of these PAs, the design of a 0.5 W GaN-based integrated Class-J PA that considers output matching network element losses for realizing on-chip output matching is presented in, ^21^ but because of the limitations of device technology and the low-Q on-chip matching network losses, its efficiency and output power are less than those of discrete PAs. An integrated Class-J PA using CMOS technology is presented in, ^22^ where the effect of knee voltage is considered to analyze 2nd harmonic losses for deriving modified design equations. However, the staked FET must be used for implementation because of the CMOS PA’s low breakdown voltage. The improvement in power output and D. E of a Class-J PA was presented in ^23^^,^^24^ by realizing a proper half-wave rectified sinusoidal waveform at the gate of the transistor. Apart from the usefulness of this method, it needs additional circuitry because of higher-order filters, which complicates the design and implementation of the PA. A methodology to improve the Class-J PA’s performance by injecting the active power at the 2 ^nd^ harmonic frequency has been proposed in, ^25^ which causes an improvement in drain efficiency, but because of doubling and filtering, the design becomes complicated, and an increase in chip area makes it less appealing for integration. Based on the literature, it is understood that among the various modes of PAs, a Class-J PA can even satisfy the enhanced bandwidth requirement of the emerging 5G smart meter/smart grid applications without sacrificing linearity and efficiency if the appropriate matching networks are designed. Therefore, in this paper, a Class-J mode PA is chosen, and a design methodology is proposed to achieve the efficiency and bandwidth requirements of 5G wireless communications of smart grids. However, the important point to be considered when designing a PA for these smart grid applications is the frequency at which it has to be operated. Hence, different frequency bands, including LTE and 5G NR (new radio), ^26^^–^^28^ are analyzed to choose the appropriate operating frequency of the proposed Class-J PA. Recently, a frequency band termed citizen broadband radio service (CBRS) with 3.5 GHz center frequency (i.e., 5G sub6 GHz frequency) was allocated for public usage. ^29^ This spectrum provides good space for 5G smart grid wireless communication applications. Hence, Initially, the proposed Class-J mode PA was designed to operate at a center frequency of 3.5 GHz (sub < 6 GHz) 5G frequency). The main contribution of this research paper involves the analysis of various matching network methods/topologies and the design of appropriate I/P and O/P matching networks to match a 50 Ω source and loads with the desired optimum source and load impedances of the transistor with respect to maximum PAE determined by load pull to achieve Class-J mode operation with the desired bandwidth of 5G smart grid specifications. The PA simulations were performed in the Advanced Design Systems (ADS) EDA tool, and this paper's structure is described as follows. A stepwise design procedure of a Class-J PA based on its theory with three approaches of lumped element-based input and output M. Ns for matching a 50 Ω source and load terminations with the transistor's optimum source and load impedances to obtain Class-J mode operation is described in the Methodology section. The schematic Class-J PA circuit simulation results as per the design methodology and their comparison with similar recent works are presented in the Results and Discussion sections. Finally, the advantages and limitations of this proposed research work are detailed in the Conclusions.

## Methods

From the theory of the Class-J operation mode introduced and developed by S.C. Cripps, ^19^ the high-efficiency amplification in broadband can be obtained by terminating the output of transistor M _1_ to appropriate fundamental (Z _f0_) and second harmonic (Z _2f0_) optimal load impedances, as shown in Equations (1) and (2), at different frequencies over the desired bandwidth, as shown in Figure 2(a).

$$Z_{f0}=\frac{\left(V_{\mathrm{DD}}-V_{\mathrm{th}}\right)\left(1+\mathrm{j\alpha}\right)}{\text{Imax}/2}=R_{\mathrm{opt}}+\mathrm{j\alpha}R_{\mathrm{opt}}$$ (1)

Z2f0=−VDD−Vthjα2Imax3π=−j3π8αRopt (2)

where R _opt_ is the optimum resistance, which can be expressed as shown in Equation (3).

$$R_{\mathrm{opt}}=2\left(V_{\mathrm{DD}}-V_{\mathrm{th}}\right)/I_{\max}$$ (3)

**Figure 1.**
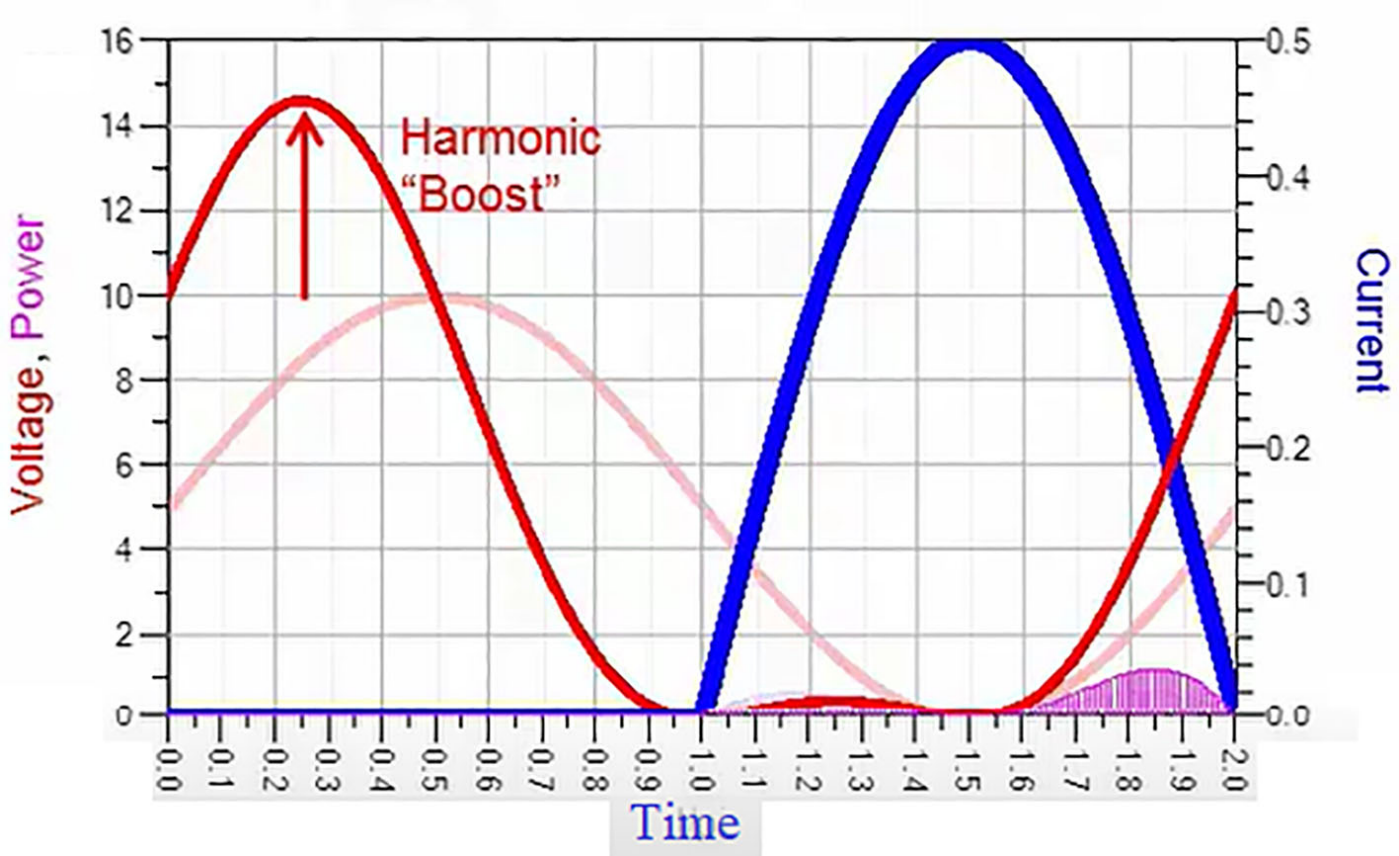
Class-J mode voltage and current waveforms.

With these optimum load impedances presented, in Figure 1, we can observe that the drain voltage (V _DS_) is boosted with a phase shift.

Thus, the phase shift and boost in drain voltage (V _DS_) cause a slight overlap with drain current (I _D_), making the Class-J power amplifier highly efficient. Although this waveform shows the feature of a switching mode PA, the Class-J mode PA can provide linearity similar to the Class-B or AB modes because of its non-switching mode of operation. Unlike in Class-B, harmonic traps are unnecessary, making it suitable for wideband 5G applications.

The various steps involved in the design methodology of the proposed Class-J PA are discussed, as illustrated in the flowchart shown in Figure 2(b).

**Figure 2(a).**
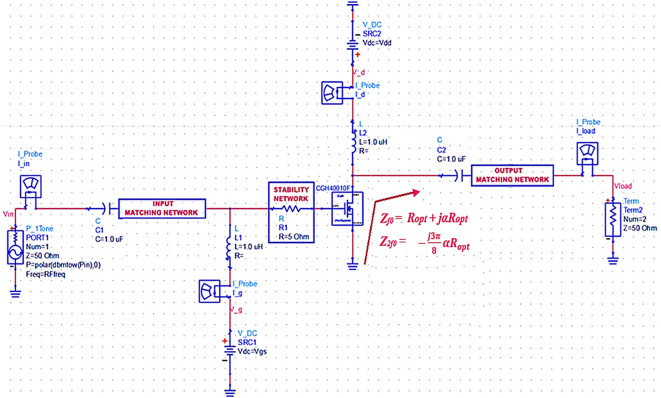
Sample Class-J mode PA topology.

**Figure 2(b).**
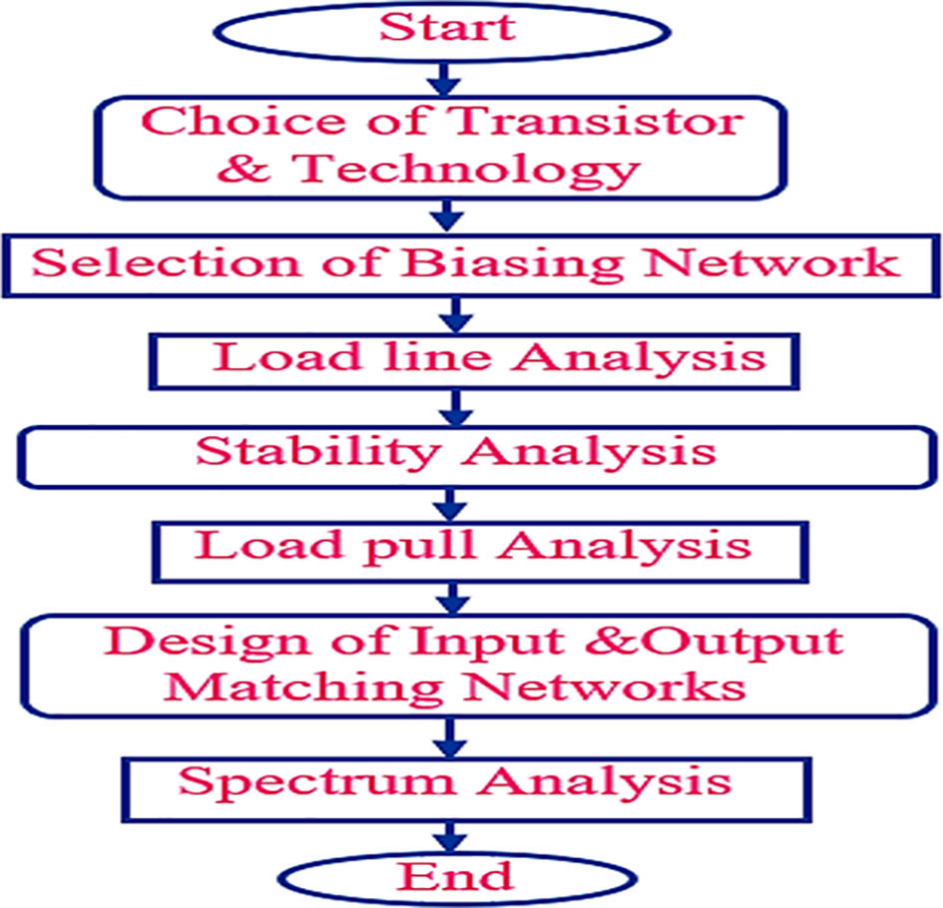
Flow chart.

### Selection of the transistor and technology

As per the trend of designing a power amplifier, the Advanced Design System (ADS) tool was used to design, and a CREE Device model (CGH40010F) based on GaN technology was used for simulation. The CGH40010F GaN transistor is chosen based on the features mentioned in its datasheet to obtain the desired power output required for smart meter applications. The gate threshold voltage (V _th_) and gate quiescent voltage (VG _Q_) of the chosen (CGH40010F) GaN transistor can also be obtained from its datasheet. To obtain a Class-J PA mode, the load impedances to be presented to this transistor (M1) (i.e., (CGH40010F) GaN transistor) are calculated theoretically using Equations (1) and (2) and can be verified using the Class-J workspace in the ADS design tool.

### Selection of the biasing Network

As per the design idea of the power amplifier for this work, the supply voltage V _DD_ = 28 V and gate bias voltage V _GG_ ≅ V _K_ (threshold voltage) (i.e., with a quiescent bias current of I _q_ = 2% of Imax) are chosen based on the (CGH40010F) GaN transistor's (M _1_) datasheet for biasing it through a fixed-bias network to operate as a Class-B PA to obtain a half-wave rectified drain current (I _D_). With the use of the fixed-bias network shown in Figure 3(a), the DC-IV characteristics of the CGH40010F GaN transistor are drawn, and the quotient (Q) or bias point is obtained by adjusting marker m2 on the load line to operate it in Class-B mode, as shown in Figure 3(b).

**Figure 3(a).**
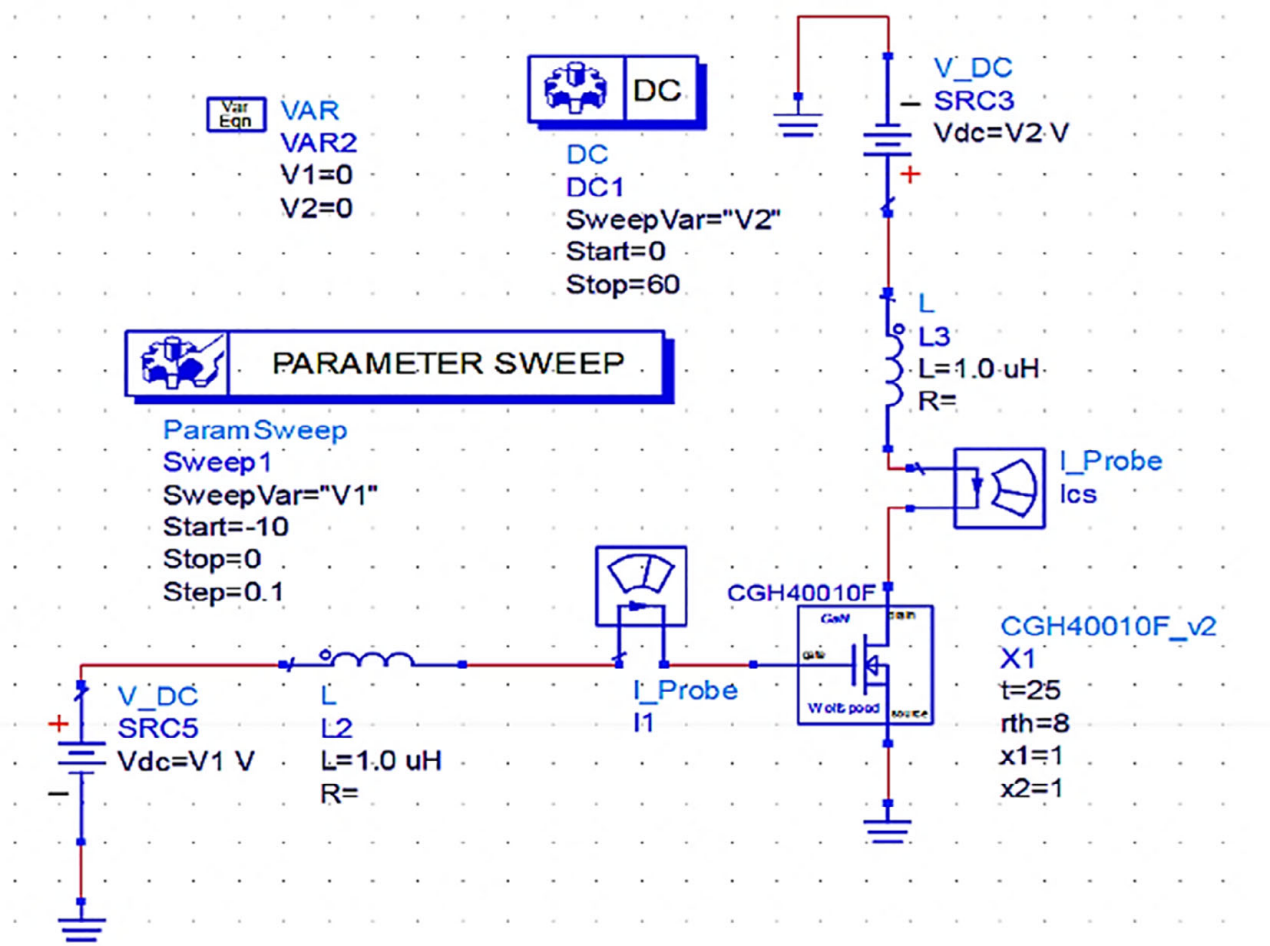
Fixed-bias network of GaN transistor.

**Figure 3(b).**
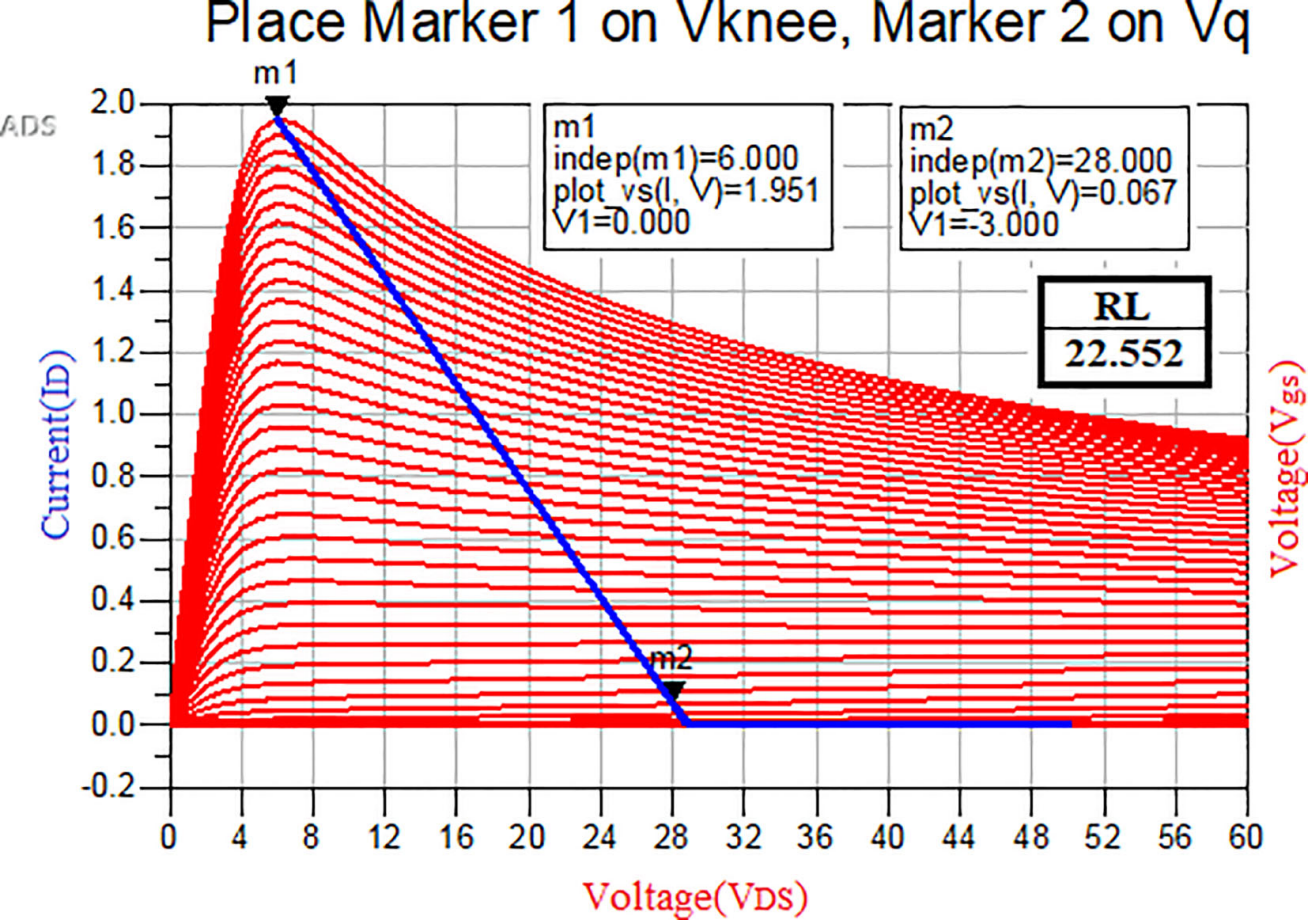
DC-IV Characteristics of GaN transistor.

### Stability analysis

After choosing the bias point of the transistor while designing the power amplifier, one of the most important considerations is that it should be unconditionally stable irrespective of the frequency under normal operating conditions. To maintain the PA’s stability at low frequencies, a stabilization circuit with a resistor connected in series with the transistor gate terminal is employed based on the datasheet of the CGH40010F GaN transistor. This series resistance can stabilize the transistor across the entire range of desired frequencies. To ensure the unconditional stability of the PA, the main conditions to be satisfied are that the Rollet stability factor is more than unity (i.e., ( *K*>1)) and the stability measure ( *b*) is positive. The Rollet stability and stability measures can be calculated theoretically by Equations (4) and (5), respectively.

$$K=\left\{1-{\left|S_{11}\right|}^{2}-{\left|S_{22}\right|}^{2}+{\left|S_{11*}S_{22}-S_{12*}S_{21}\right|}^{2}\right\}/\left\{2*\left|S_{12*}S_{21}\right|\right\}$$ (4)

$$b=\left\{1+{\left|S_{11}\right|}^{2}-{\left|S_{22}\right|}^{2}-{\left|S_{11*}S_{22}-S_{12*}S_{21}\right|}^{2}\right\}$$ (5)

**Figure 4.**
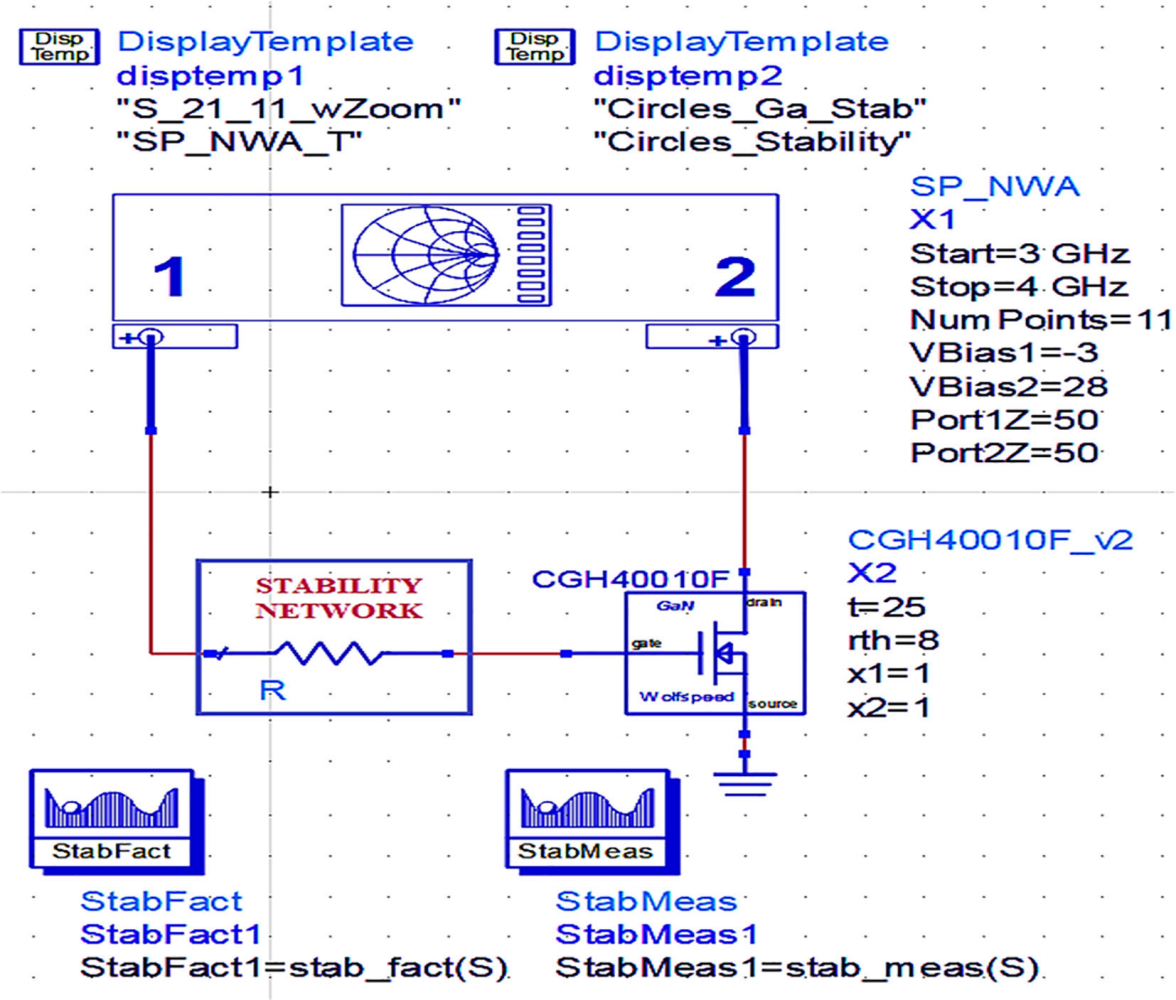
Stability analysis of a GaN transistor using Network Analyzer.

However, practically unconditional stability of the PA can be ensured using Network Analyzer for S-Parameters (SP_NWA) from simulation instrument components, and the stability factor and its variation w.r.t. frequency can be obtained with the use of measurement expression functions and data display templates in Advanced Design System (ADS) tool, as shown in Figure 4.

### Selection of optimum input and output impedances of the transistor

Generally, the transistor's optimum input and output impedances used for a PA design can be obtained by conducting load-pull simulations with the reference source and load impedances chosen from its datasheet.

As we are designing a Class-J PA in this research work, the reference source impedance can be chosen from the transistor's datasheet (if not mentioned in the datasheet, we can take approximately (5+j*0 Ω) for any GaN device). The reference fundamental and second harmonic impedances can be calculated theoretically using Equations (1), (2), and (3).

The reference target optimum impedances required to obtain Class-J operation can be determined using the Class-J ADS workspace utility, which is developed with mathematical design equations based on the load line.

**Figure 5.**
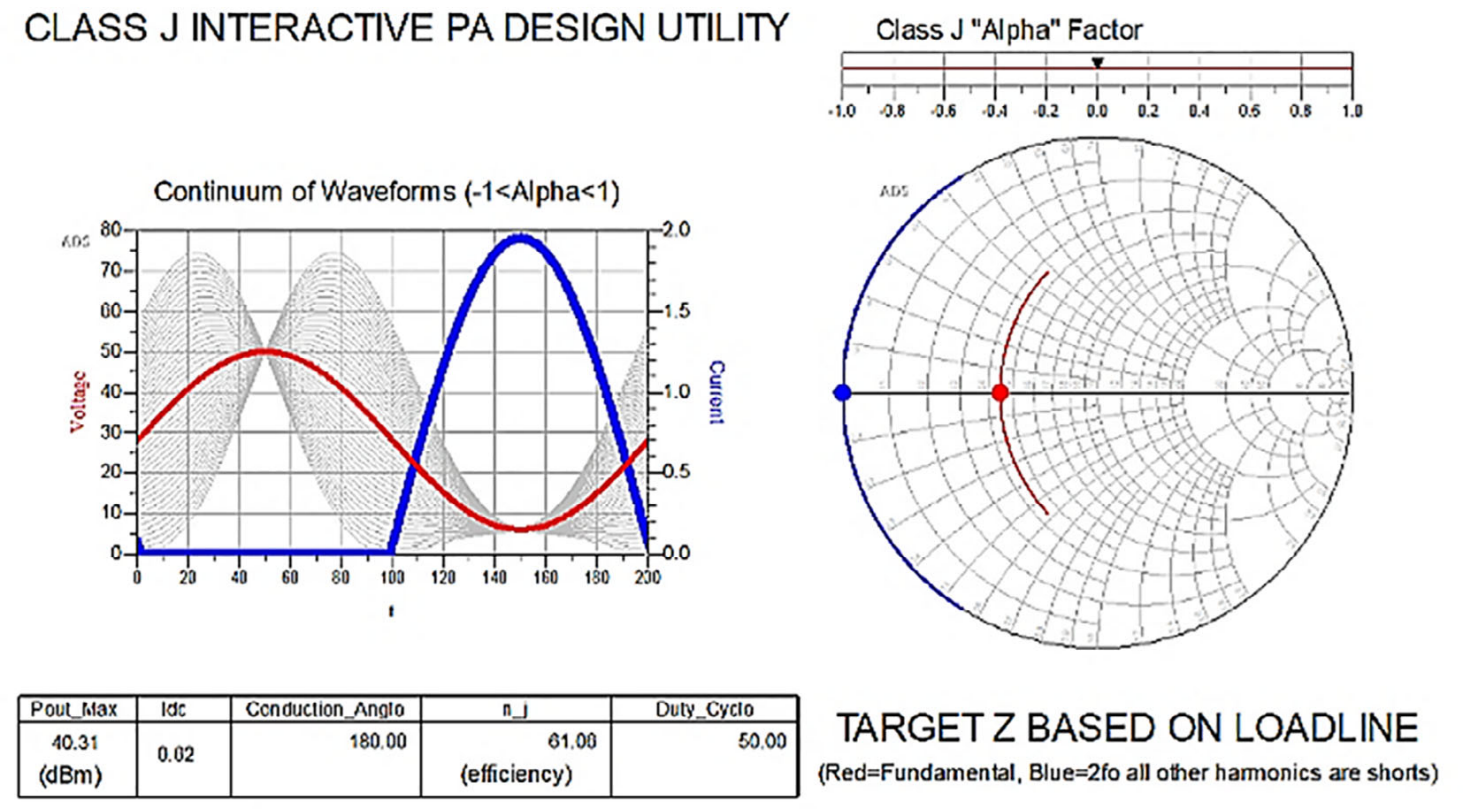
Class-B operation mode with (α = 0).

After choosing the bias (Q) point by adjusting the load line on DC-IV characteristics, which are obtained from the fixed-bias network shown in Figure 3(b), and by keeping the Alpha (α) factor in Equations (1) and (2) as “zero (0)” on the slider, as shown in Figure 5, the fundamental is terminated to a resistive load and higher-order harmonics are terminated as short, which leads to the Class-B mode of operation.

**Figure 6.**
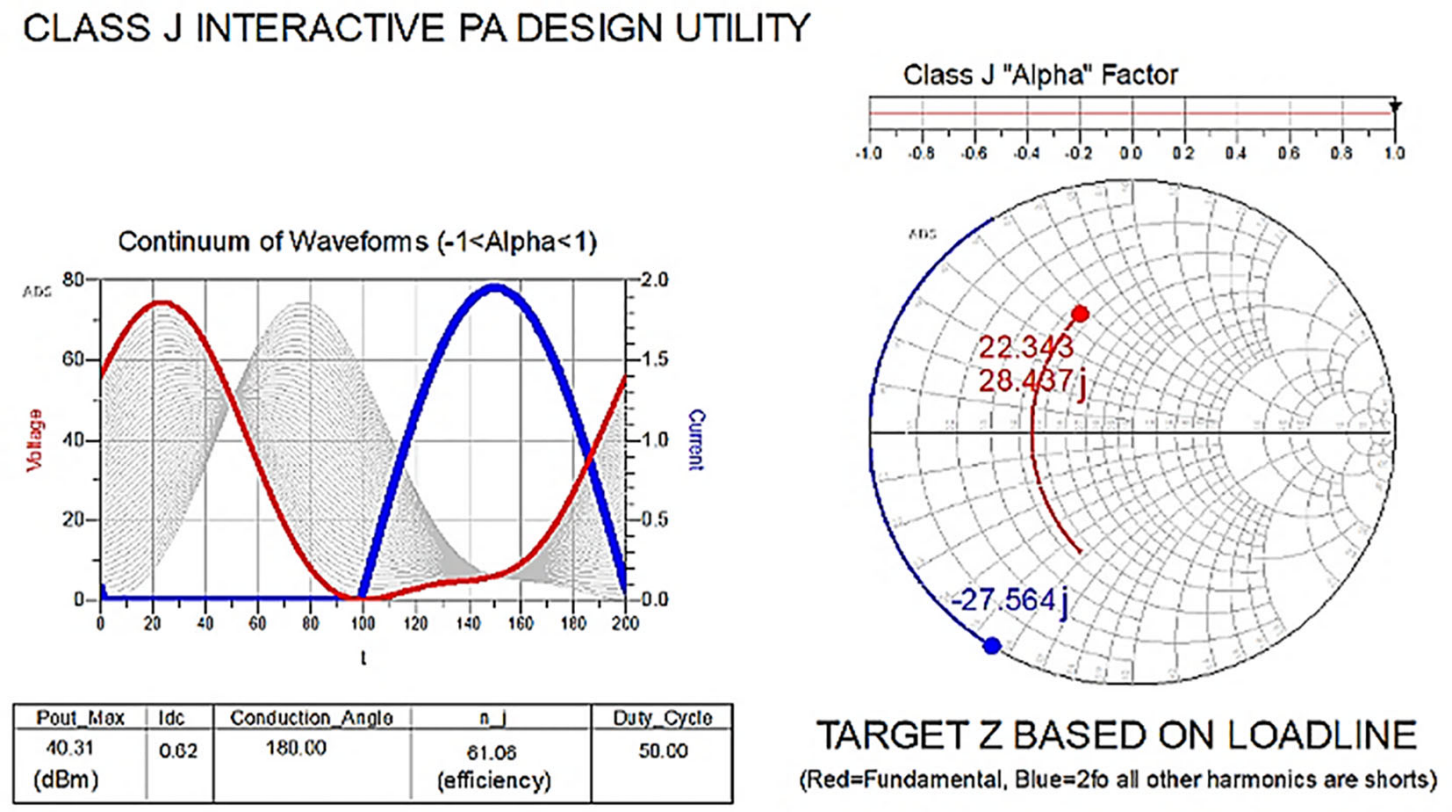
Class-J operation mode with (α = 1).

The reference target optimum impedances required for Class-J operation can be obtained by moving the alpha factor (α) in Equations (1) and (2) from 0 to 1 on the slider in Figure 6. With this target fundamental and second harmonic load impedance, the drain voltage (V _DS_) is boosted with a phase shift.

The transistor’s drain voltage can be expressed theoretically as shown in Equation (6) from which the optimum impedances as shown in Equations (1) and (2) that are required for Class-J operation are obtained by considering the effect of the threshold voltage.

$$V_{J}\left(\theta \right)=V_{\mathrm{dc}}\left(1+\text{sinθ}\right)\left(1+\text{αcosθ}\right)$$ (6)

The “Alpha” factor (α) as shown in Equation (6) can be varied over a range from -1 to 1. Because each drain voltage waveforms for different values of (α) when combined with half-rectified current results in the same theoretical efficiency, causing the Class-J mode to expand its BW.

When α in Equation (6) changed from “0” to “1” it can be expanded as shown in Equation (7).

$$V_{J}\left(\theta \right)=V_{\mathrm{dc}}\left(1+\text{sinθ}+\text{cosθ}+\frac{1}{2}\sin2\theta \right)$$ (7)

The addition of the *sinθ* and *cosθ* functions in Equation (7) causes a phase shift to the drain voltage at fundamental frequency and the product term *sin*2 *θ* causes a second harmonic voltage that adds in phase to boost the fundamental, which leads to the harmonic boost in the drain voltage as shown in Figure 6.

### Load-pull analysis

**Figure 7.**
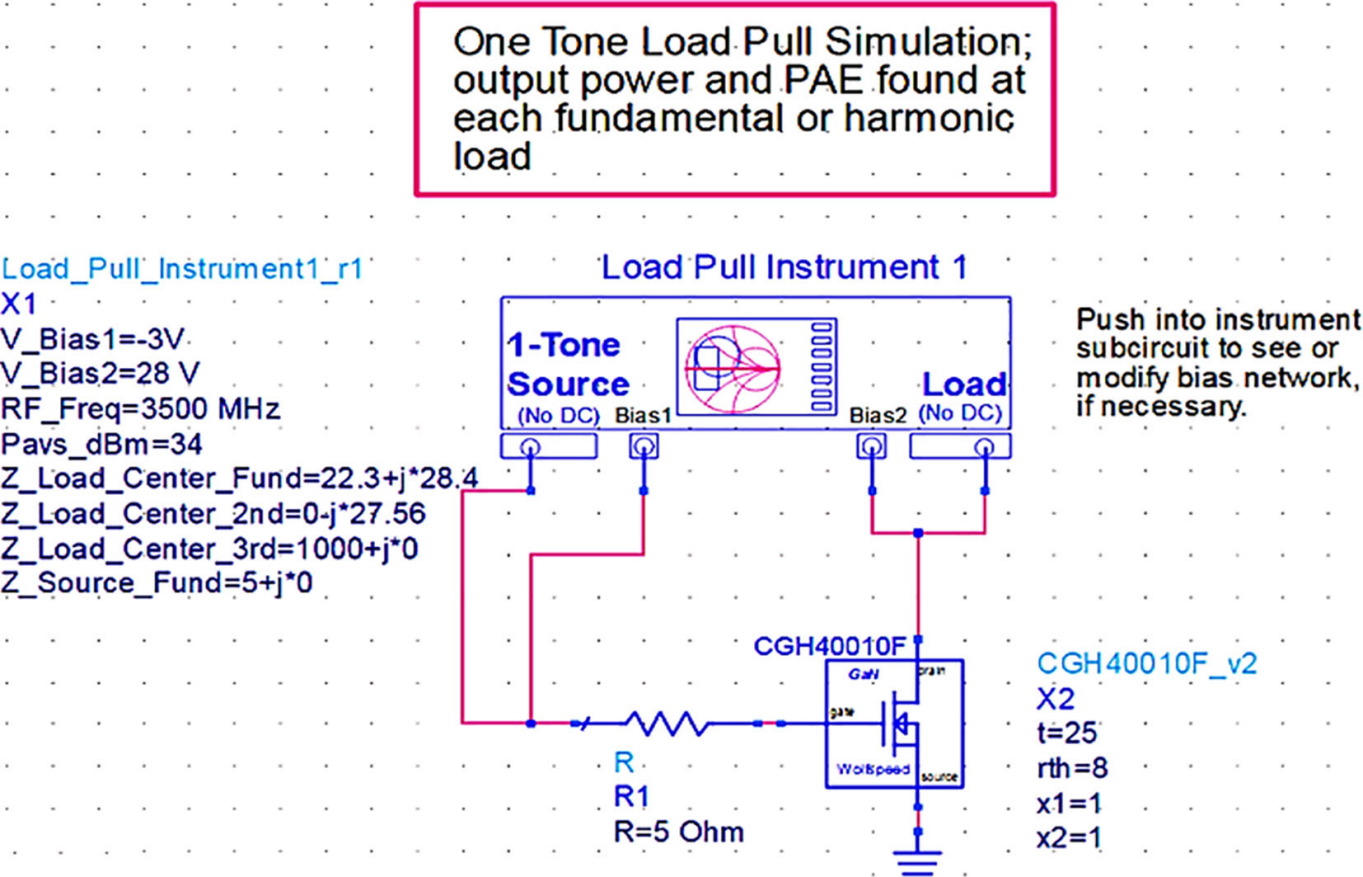
Load-pull analysis of the stabilized transistor.

To verify the target fundamental and second harmonic load impedances shown in Figure 6, which are obtained based on the load line, load-pull simulations on the stabilized transistor must be conducted by taking them as reference impedances using a one-tone load-pull instrument at constantly available source power in the ADS EDA tool, as shown in Figure 7.

### Validation of optimum impedances obtained from load pull

**Figure 8.**
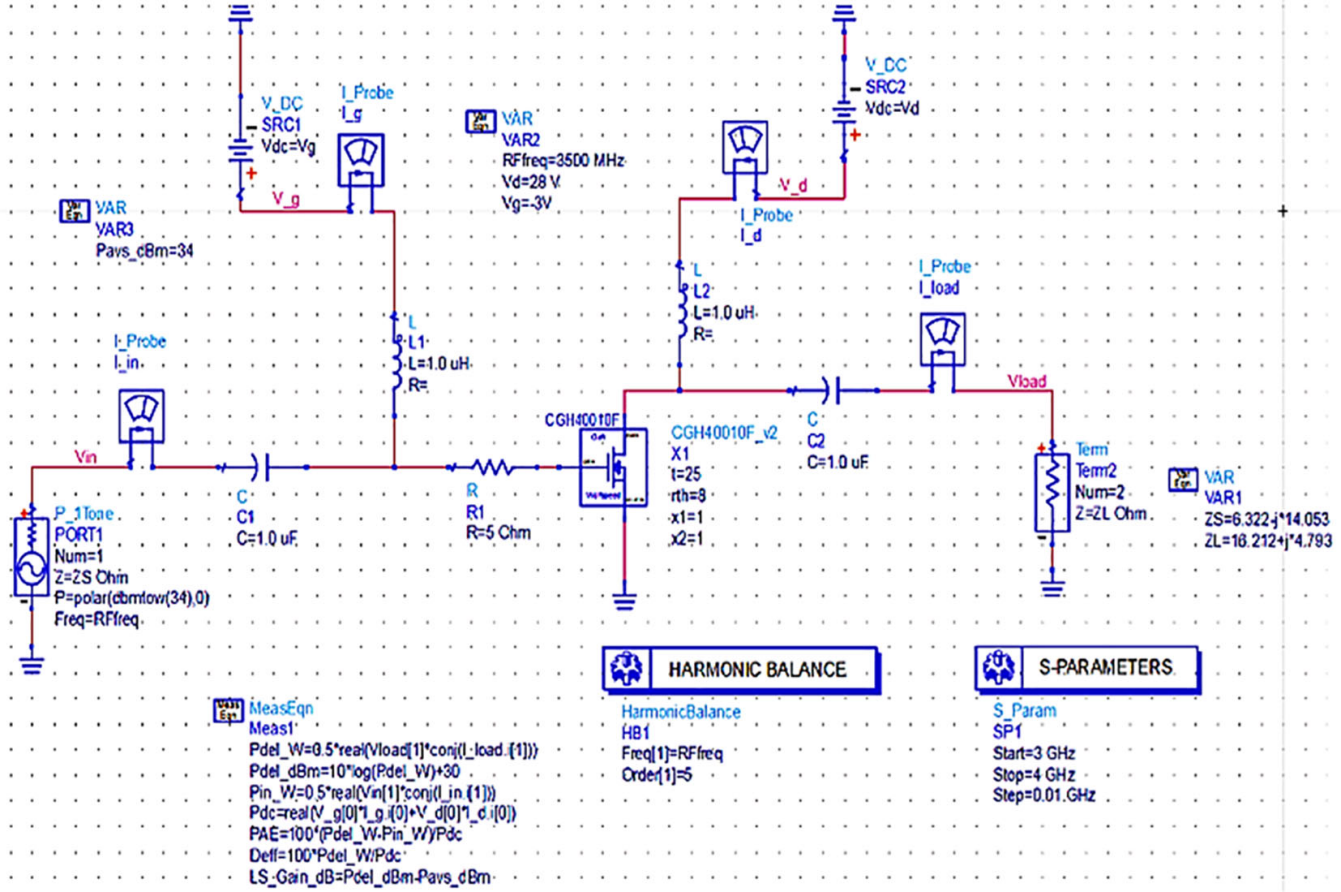
Validation of optimum impedances obtained from load-pull simulations.

The proposed Class-J PA of this work is intended to be used in 5G wireless networks in AMI/smart metering applications. The reduction of energy consumption is the key consideration while implementing the proposed Class-J PA in such applications and it mainly depends on the PAE of the proposed PA. Therefore, in this work to design the matching networks of the proposed Class-J PA the optimum impedances corresponding to its PAE are considered rather than power output. Therefore, after obtaining the optimum source, fundamental and second harmonic load impedances corresponding to the MAX PAE from the LOADPULL simulations, corresponding to the MAX PAE, they can be validated by presenting them directly to the transistor (Z _S_ and Z _L_) instead of 50 Ω termination at the source and load terminals, as shown in Figure 8.

### Input and output matching network design

After validation of the optimum input and output impedances (Z _S_ and Z _L_), the next important step in the design is the realization of the input and output M. Ns to match them with the 50 Ω termination source and load terminals. As we use the ADS EDA tool for this research work, impedance matching networks can be designed using three methods: Smith chart utility, impedance match utility, and equation-based lumped element L and π-type matching networks.

Initially, to design the input matching network, the Smith chart component (DA_smithchart1) is terminated with a source impedance of 50 Ω and the output impedance as the source impedance of the CGH40010F GaN transistor obtained from load-pull analysis. As the M. N needs to be designed at an operating frequency of 3.5 GHz, an S-parameter sweep is set up for the range of 3–4 GHz, as shown in Figure 9(a).

**Figure 9(a).**
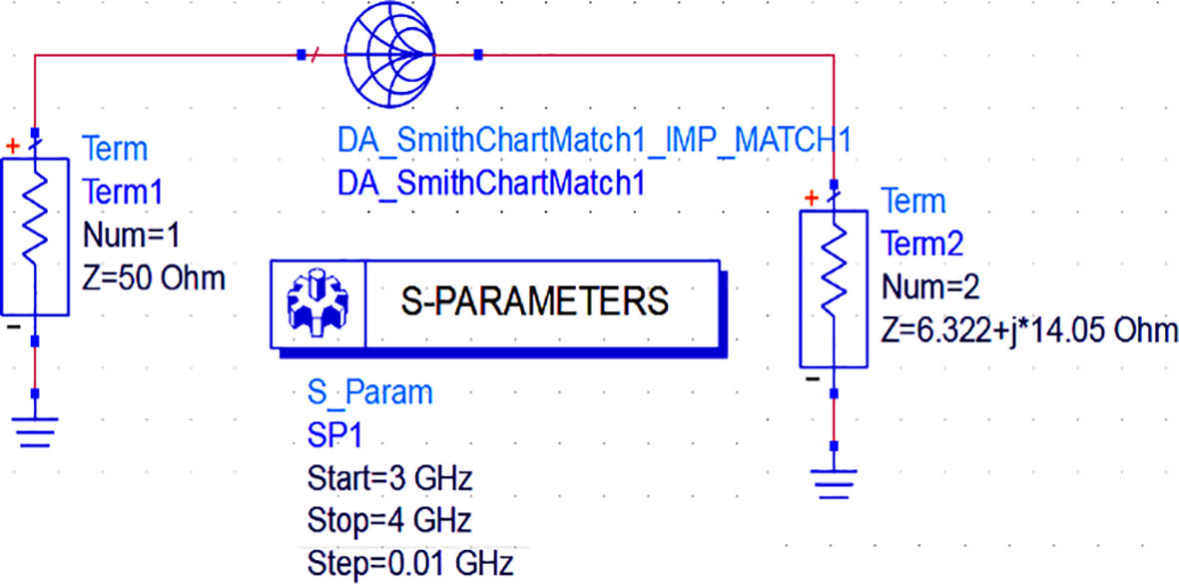
Input matching S-parameter sweep.

After setting up the S-parameter sweep, the source impedance is set as 50 Ω. The load impedance is set as the input impedance of the CGH40010F GaN transistor (i.e., obtained from the load-pull simulations) on the Smith chart utility, and the travel path from source impedance to the load impedance leads to an L-type input M. N, as shown in Figure 9(b).

**Figure 9(b).**
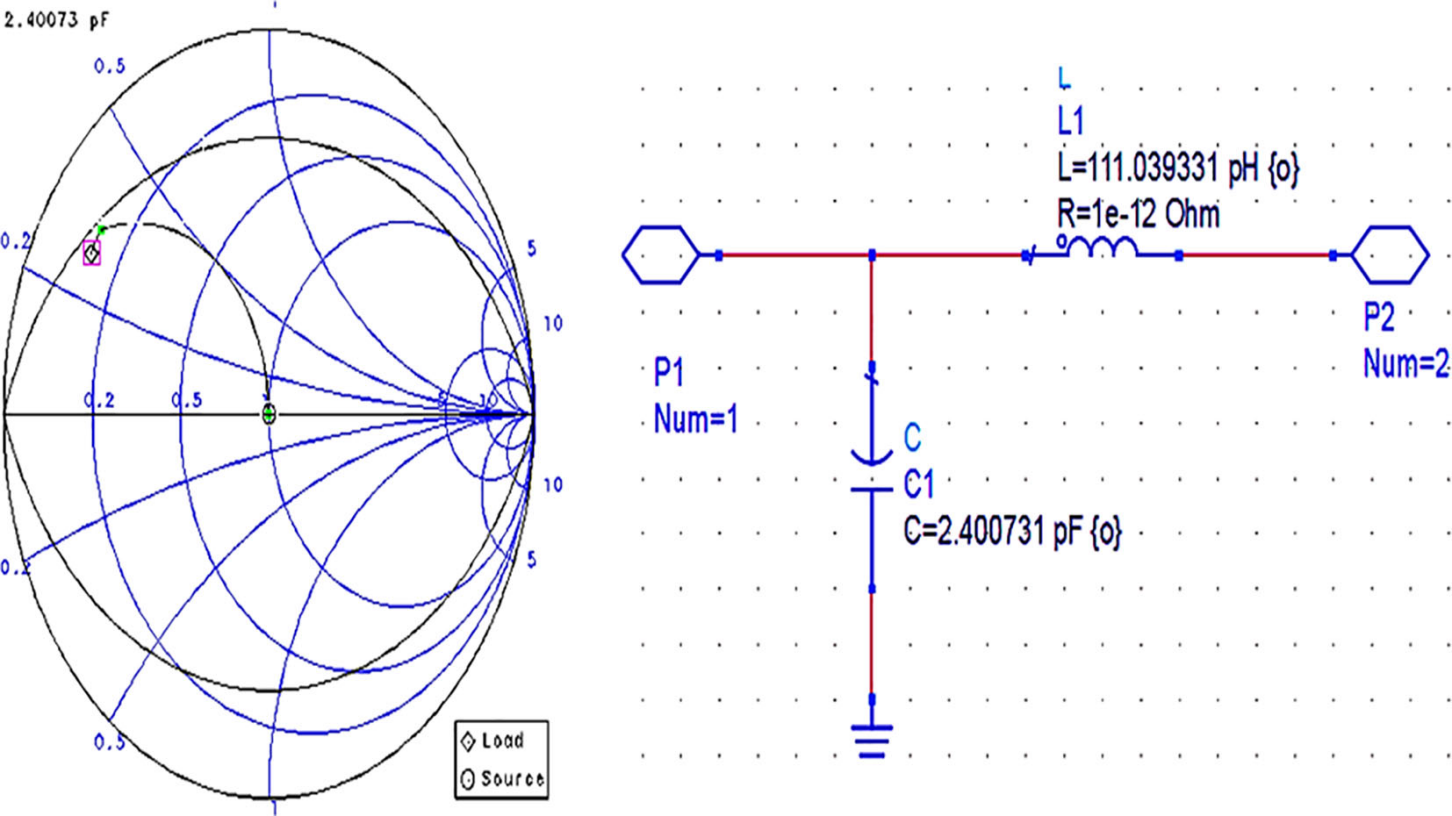
Input impedance matching network.

The output M. N is designed in the same manner as the input M.N. Nevertheless, the Smith chart component (DA _smithchart1) is terminated with source impedance as the output impedance of the CGH40010F GaN transistor (i.e., obtained from the load-pull analysis). The load is 50 Ω, and the S-parameter sweep is set up as an input matching network for the range of 3–4 GHz, as shown in Figure 10(a).

**Figure 10(a).**
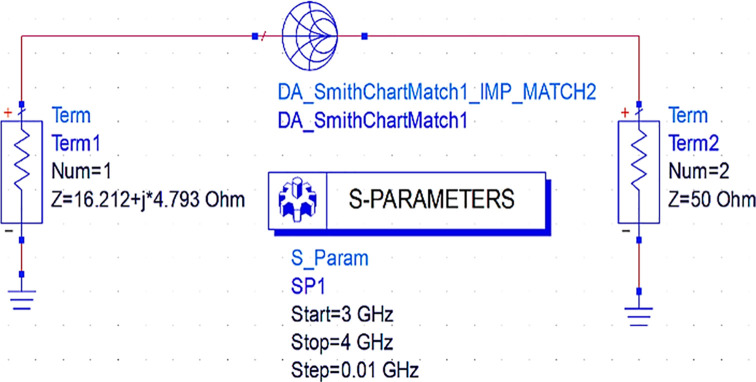
Input matching S-parameter sweep.

After setting up the operating frequency, source, and load impedances on the Smith chart utility, the travel path from source impedance to load impedance leads to an L-type output matching network, as shown in Figure 10(b).

**Figure 10(b).**
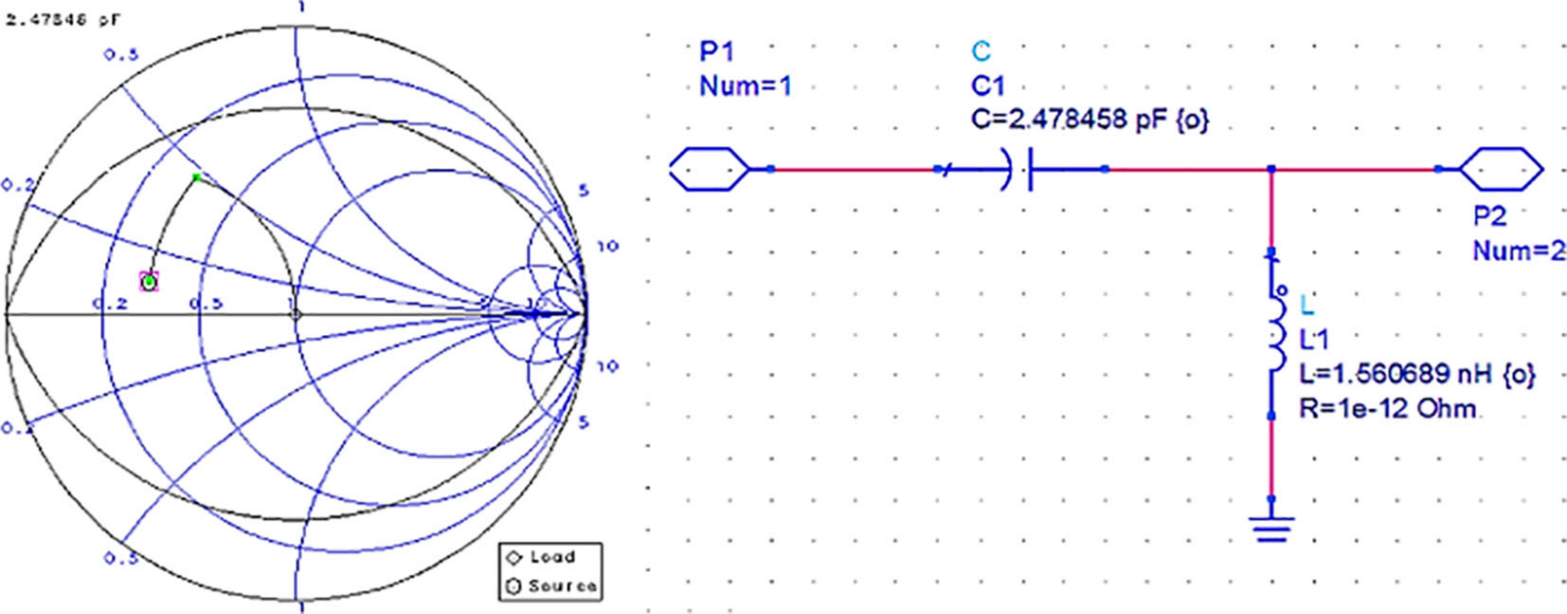
Output impedance matching network.

Initially, these matching networks are designed for a bandwidth of approximately 1 GHz with an operating frequency of 3.5 GHz (i.e., sub6 GHz). With the desired bandwidth and center frequency, the quality factor can be calculated as Q = f/BW. In Smith chart utility, this Q factor can be represented as Q circles. For this work, the M. Ns at input and output are designed with a Q-circle of 3.

Next, the matching networks to match the same input and output impedances (based on load-pull analysis) are represented using the Z2P_Eqn file with the 50 Ω source, and load terminations are designed using the L.C. bandpass match smart component using an impedance matching utility, as shown in Figure 11(a).

**Figure 11(a).**
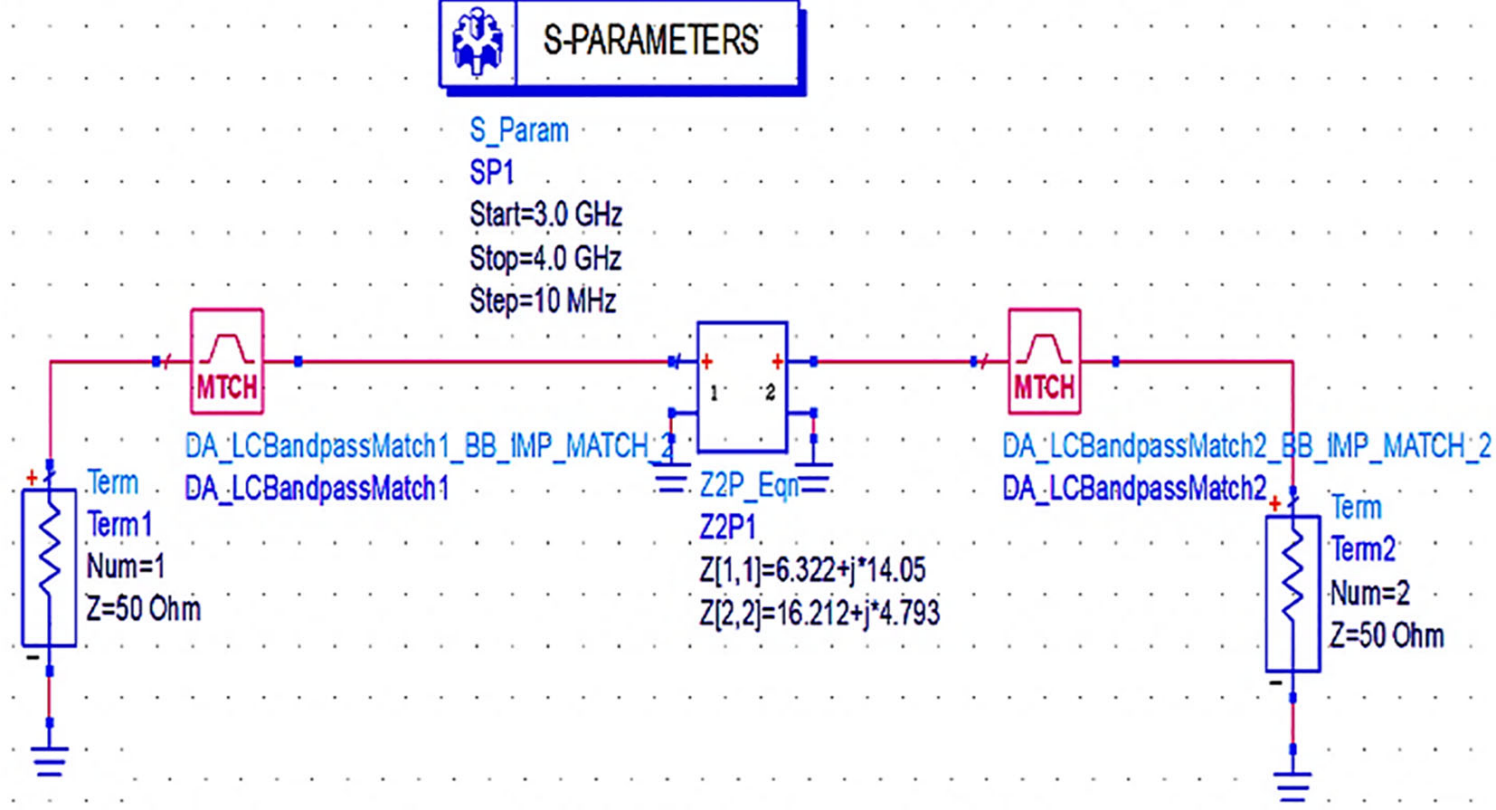
L.C. bandpass-based input and output impedance matching S-parameter sweep setup.

After setting up the range of frequencies (i.e., 3-4 GHz), the source and load impedances of approximately 10–14 matching network topologies are designed for input and output matching in impedance matching utility, from which the network topologies with fewer passband errors after optimization are chosen, as shown in Figures 11(b) and 11(c).

**Figure 11(b).**
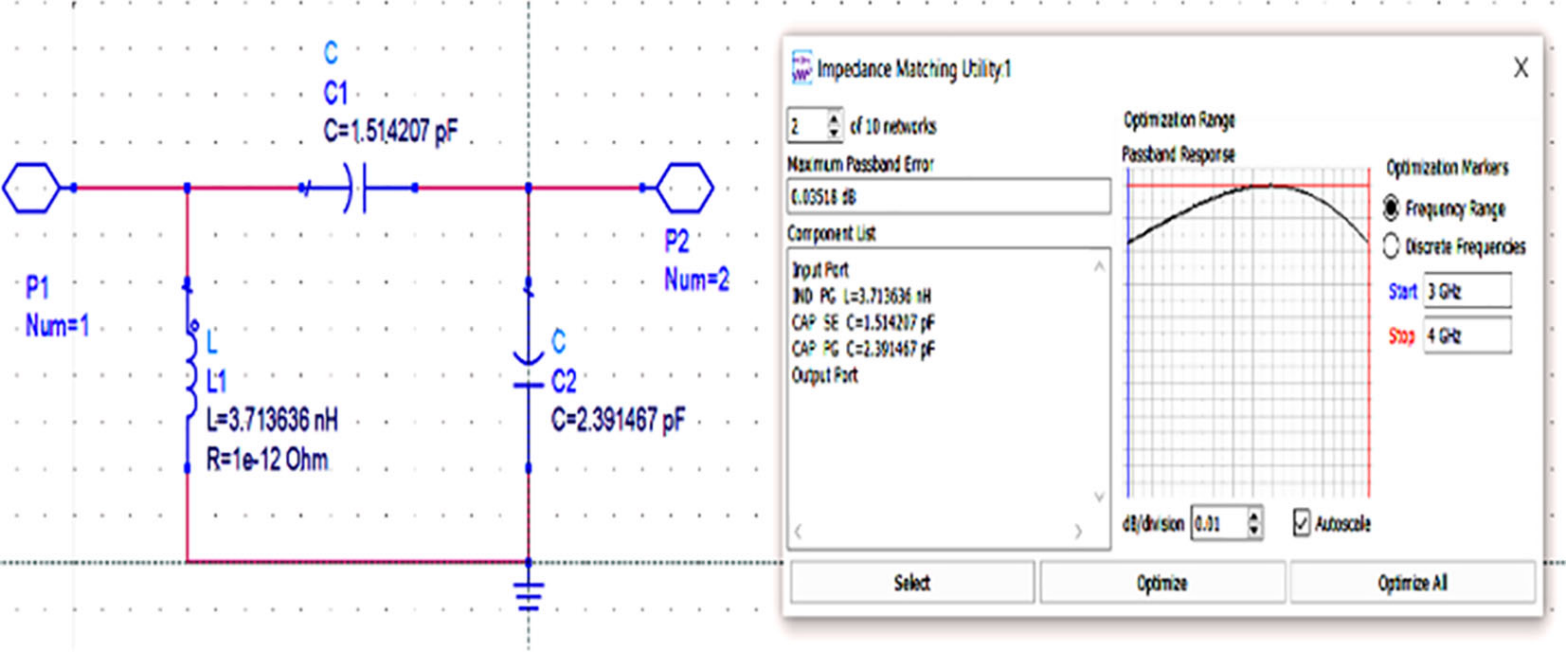
L.C. bandpass-based input matching network.

**Figure 11(c).**
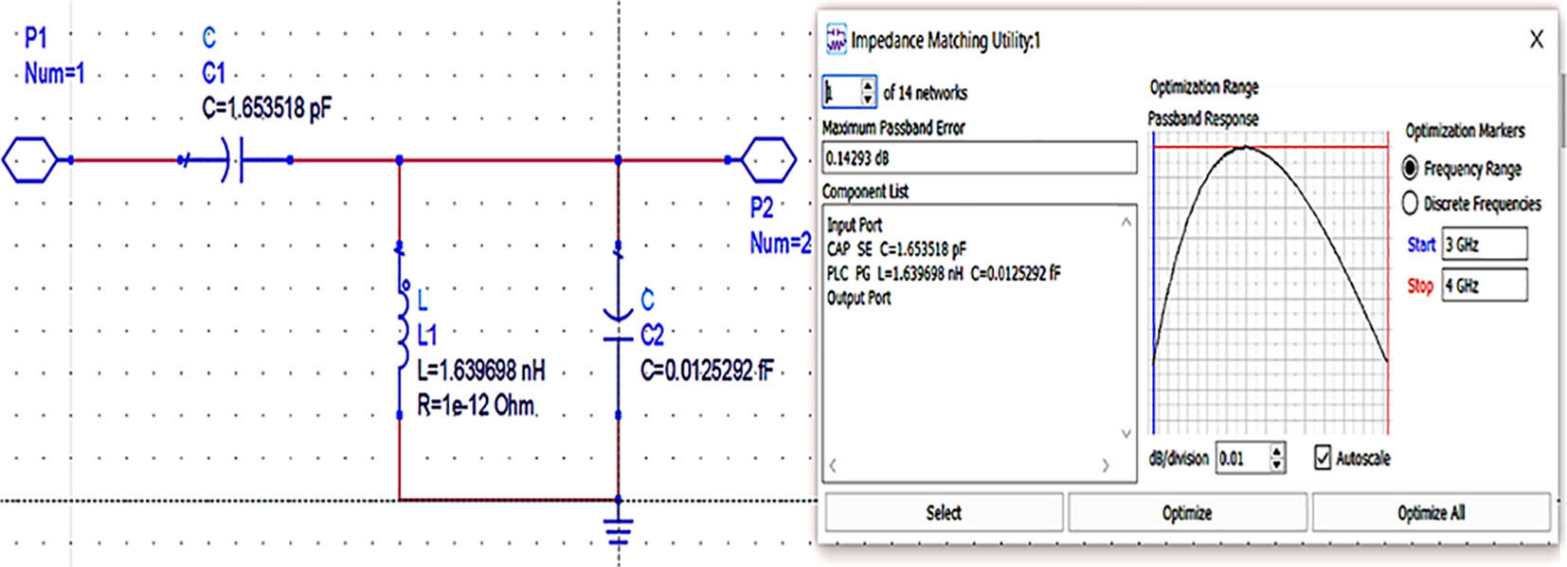
L.C. bandpass-based output matching network.

**Figure 12.**
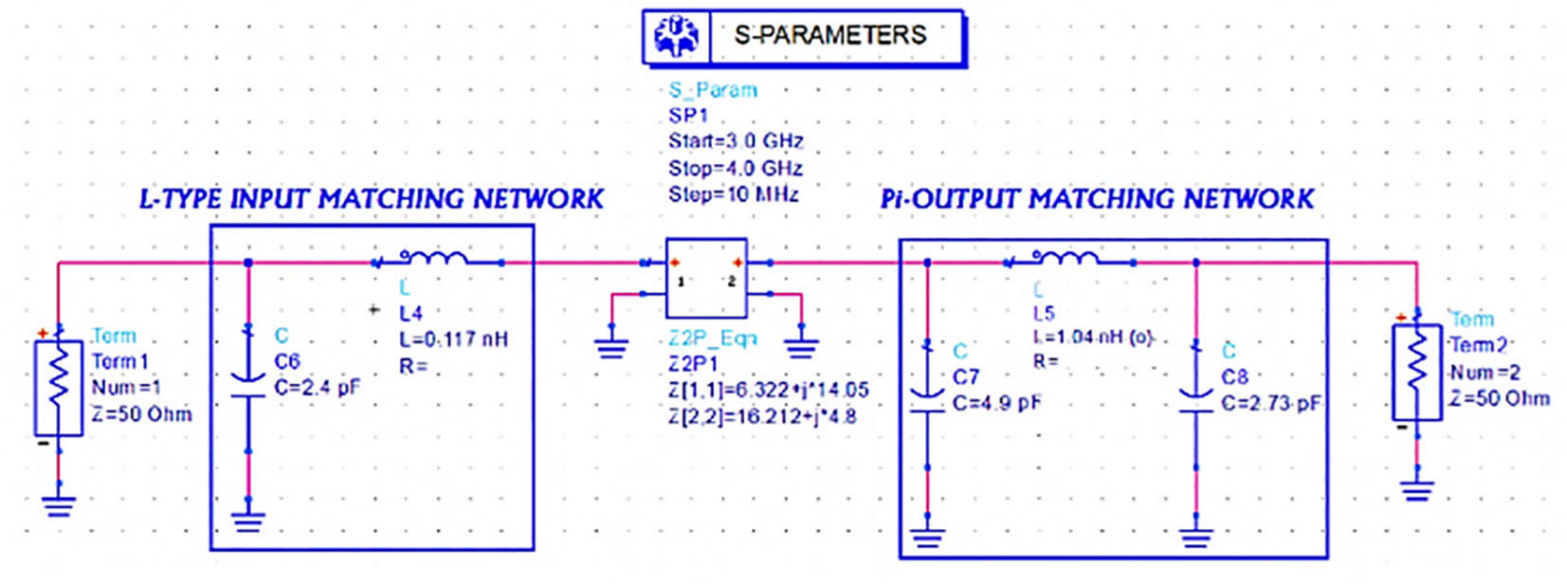
L-type input and π-type output matching networks S-parameter sweep setup.

Later, lumped element L-type input and π-type output M. Ns to match the same input and output impedances (obtained from load-pull simulations) with the 50 Ω source and load terminations are designed using basic L-type and π-type impedance matching network design equations with an operating frequency of 3.5 GHz and a Q-factor of 3 by setting up an S-parameter sweep for the range of 3–4 GHz, as shown in Figure 12.

**Figure 13.**
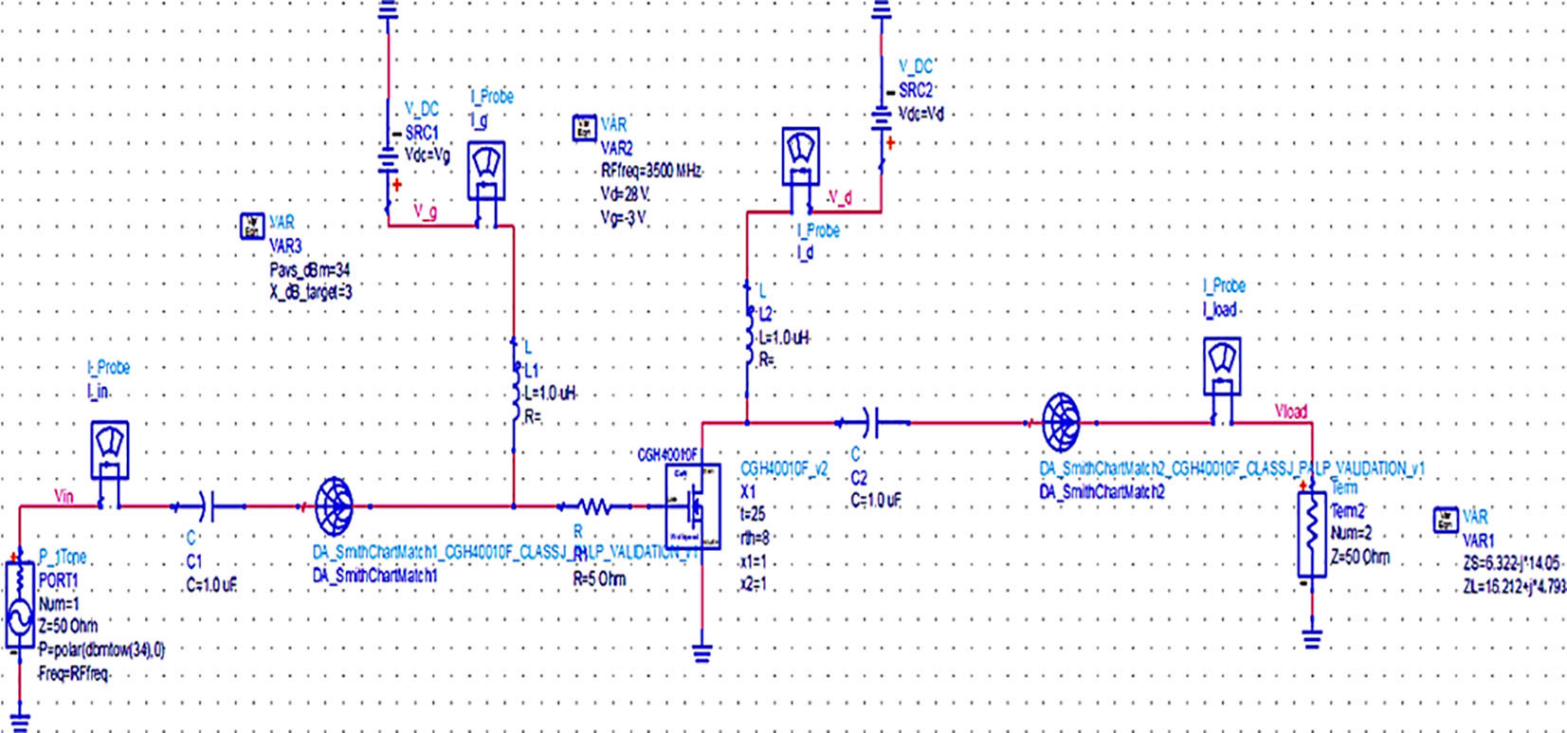
Schematic circuit of the Class-J PA with Smith chart utility-based matching networks.

**Figure 14.**
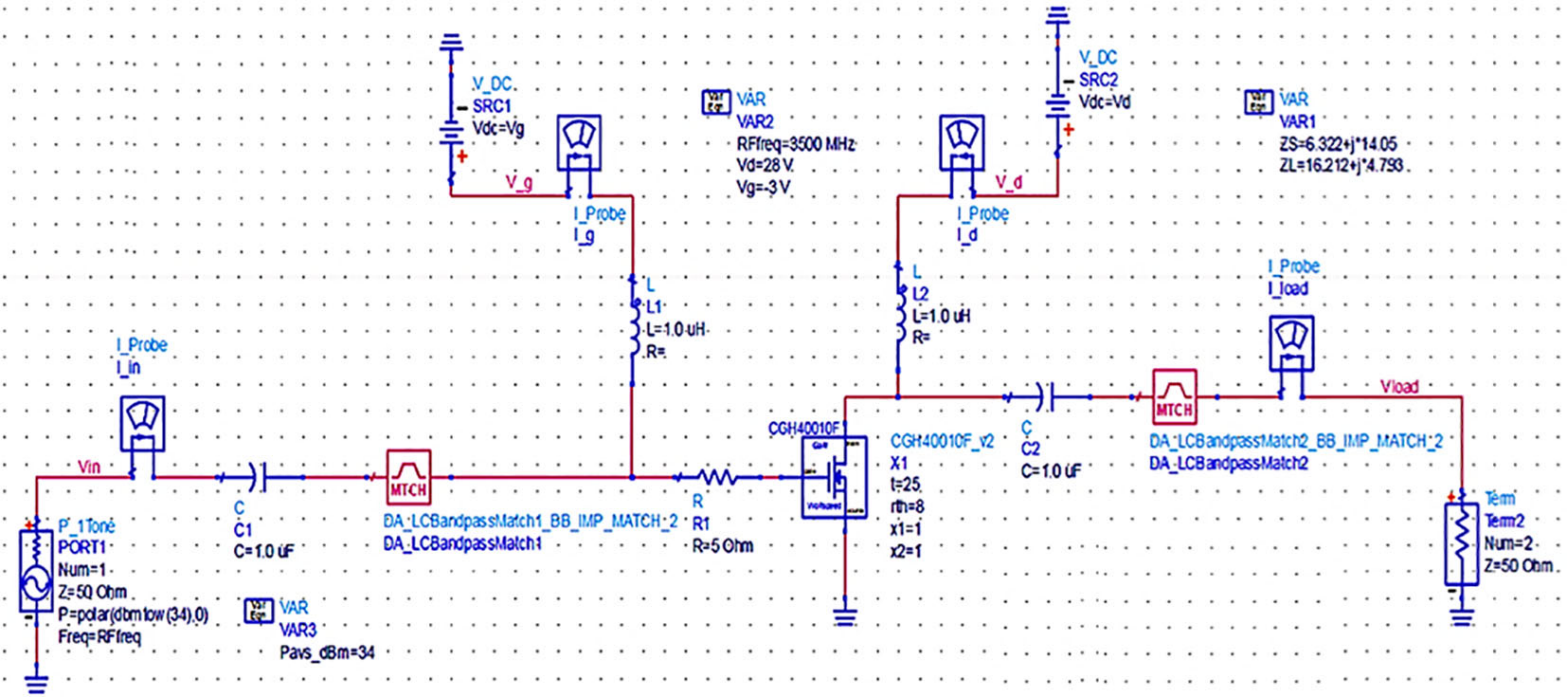
Schematic circuit of the Class-J PA with optimized LC-Bandpass matching networks.

**Figure 15.**
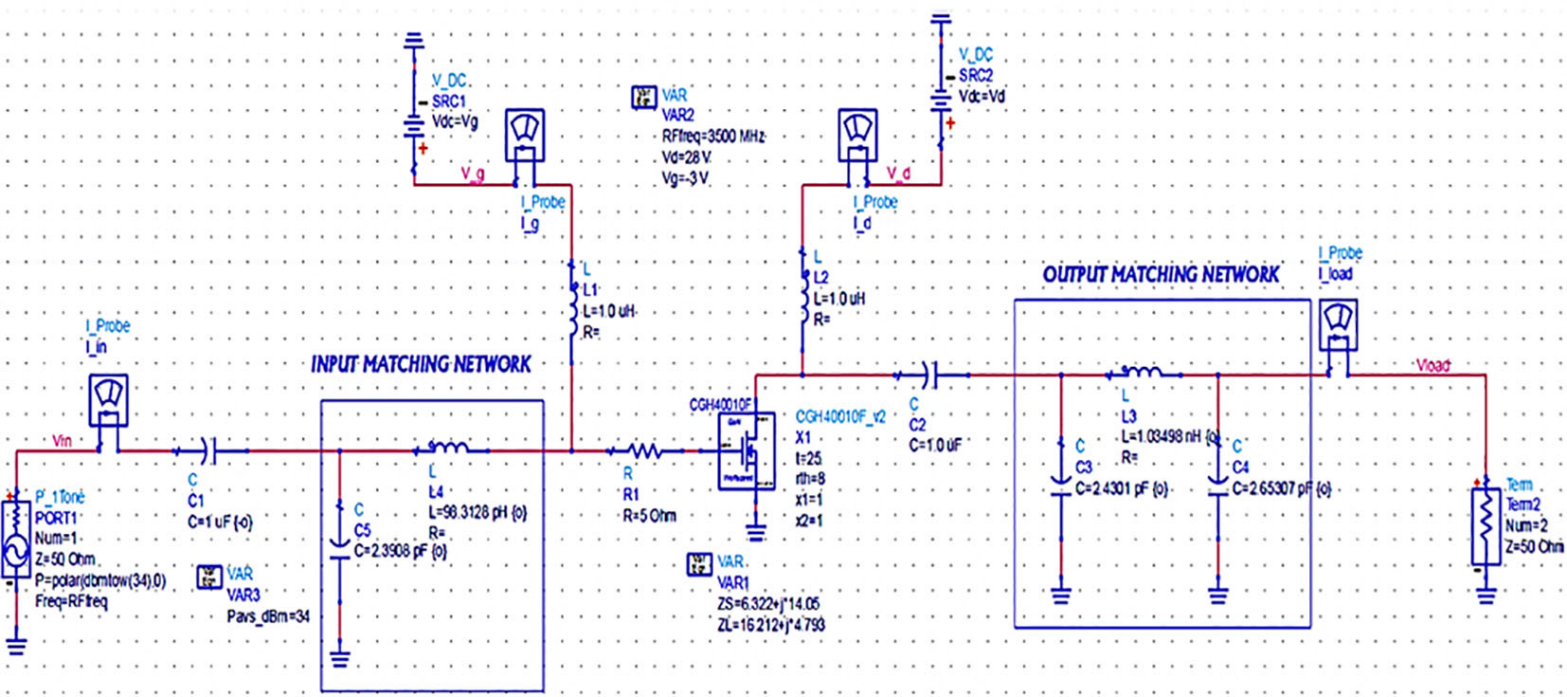
Schematic circuit of the Class-J PA with L- and π-type matching networks.

Once the matching networks are designed, all topologies are placed at the input and output of the stabilized transistor to match the optimum Z _S_ and Z _L_ with the 50 Ω termination at the source and load terminals to complete the PA design, as shown in Figures 13, 14 and 15, respectively.

The output parasitic capacitance C _DS_ of the transistor at higher-order harmonics is considered a short circuit. Once the OMN is designed, the capacitive reactance to the load-line resistance ratio [X _CDS_/R _L_] needs to be calculated. Suppose this ratio is (<=) 1; then, the matching network design is considered ideal. However, this ratio can also be above unity based on the technology and frequency of the device used in the design. As the GaN transistor is used as the main active device in the proposed Class-J PA, the second harmonic impedance condition is satisfied by its intrinsic drain-source capacitance (C _DS_) and the capacitors in its π-type OMN.

The proposed Class-J PA is designed and optimized to minimize harmonic distortion and enhance the PAE and Drain Efficiency across desired BW using load pull techniques and careful design of its M.Ns. In addition, the GaN transistor used in the Class-J PA design can deliver high output power and efficiency due to its high-power density, while maintaining linearity, which is critical for harmonic distortion control. In this work, the lower harmonic distortion of the proposed Class-J PA is validated by measuring its linearity.

## Results

**Figure 16.**
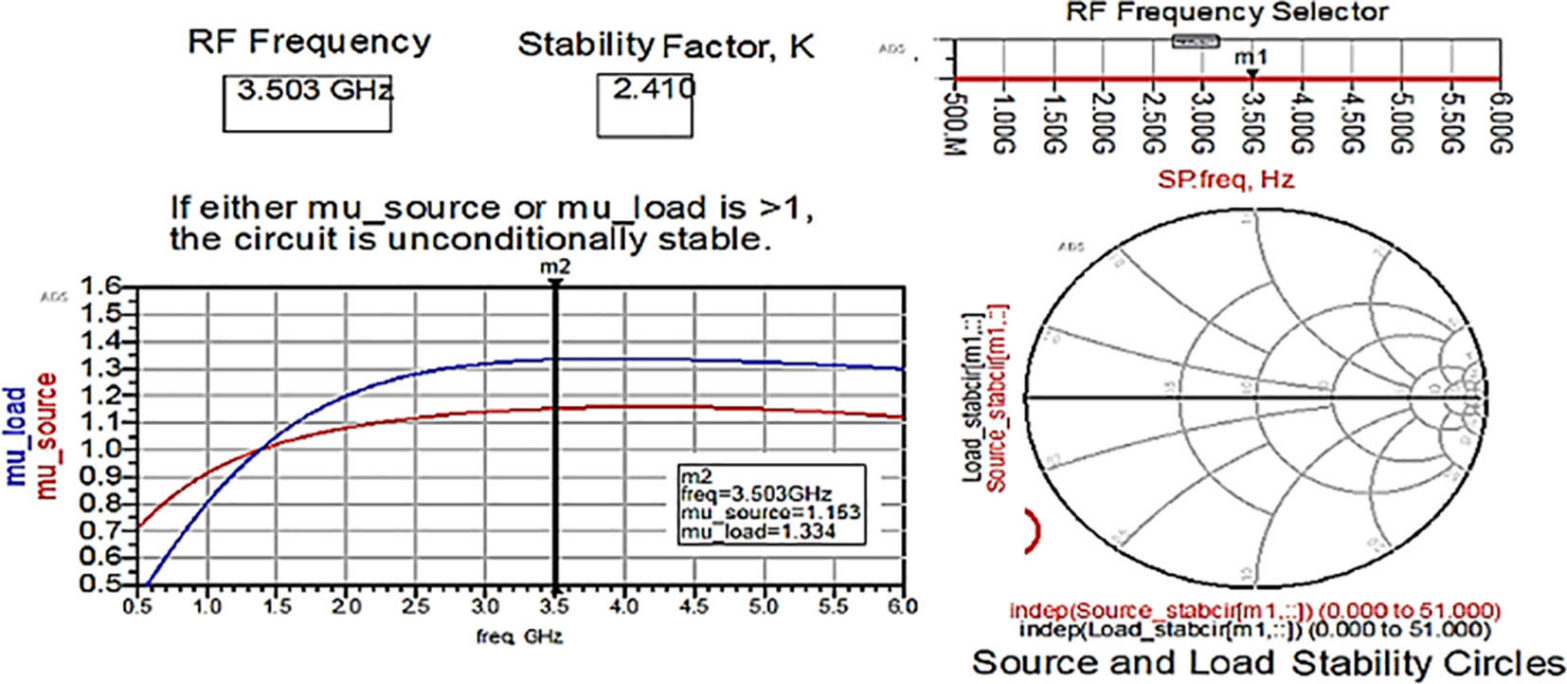
Stability factor analysis.

The Class-J PA is designed using a CGH40010F transistor with GaN technology in the Advanced Design System (ADS) EDA tool. Initially, after obtaining the bias (Q) point from the D.C. analysis, the stability analysis is performed on the CGH40010F GaN transistor with an S.P. network analyzer circuit, as shown in Figure 4. The unconditional stability of the device over the desired range of frequencies can be confirmed by checking the result on the stability factor and measuring the analysis represented using Figure 16 and Table 1. It is observed that the stability factor is >1, and the stability measure is >0, which ensures that the GaN device is unconditionally stable over the desired frequency range, i.e., (3–4) GHz.

**Table 1.** Stability analysis of the stabilized GaN transistor for the (3–4) GHz frequency range.

Freq	Stab Fact 1	Stab Meas 1
3.000 GHz	2.111	1.207
3.100 GHz	2.174	1.196
3.200 GHz	2.234	1.185
3.300 GHz	2.294	1.174
3.400 GHz	2.352	1.164
3.500 GHz	2.408	1.154
3.600 GHz	2.463	1.145
3.700 GHz	2.515	1.135
3.800 GHz	2.565	1.127
3.900 GHz	2.613	1.118
4.000 GHz	2.659	1.111

**Figure 17.**
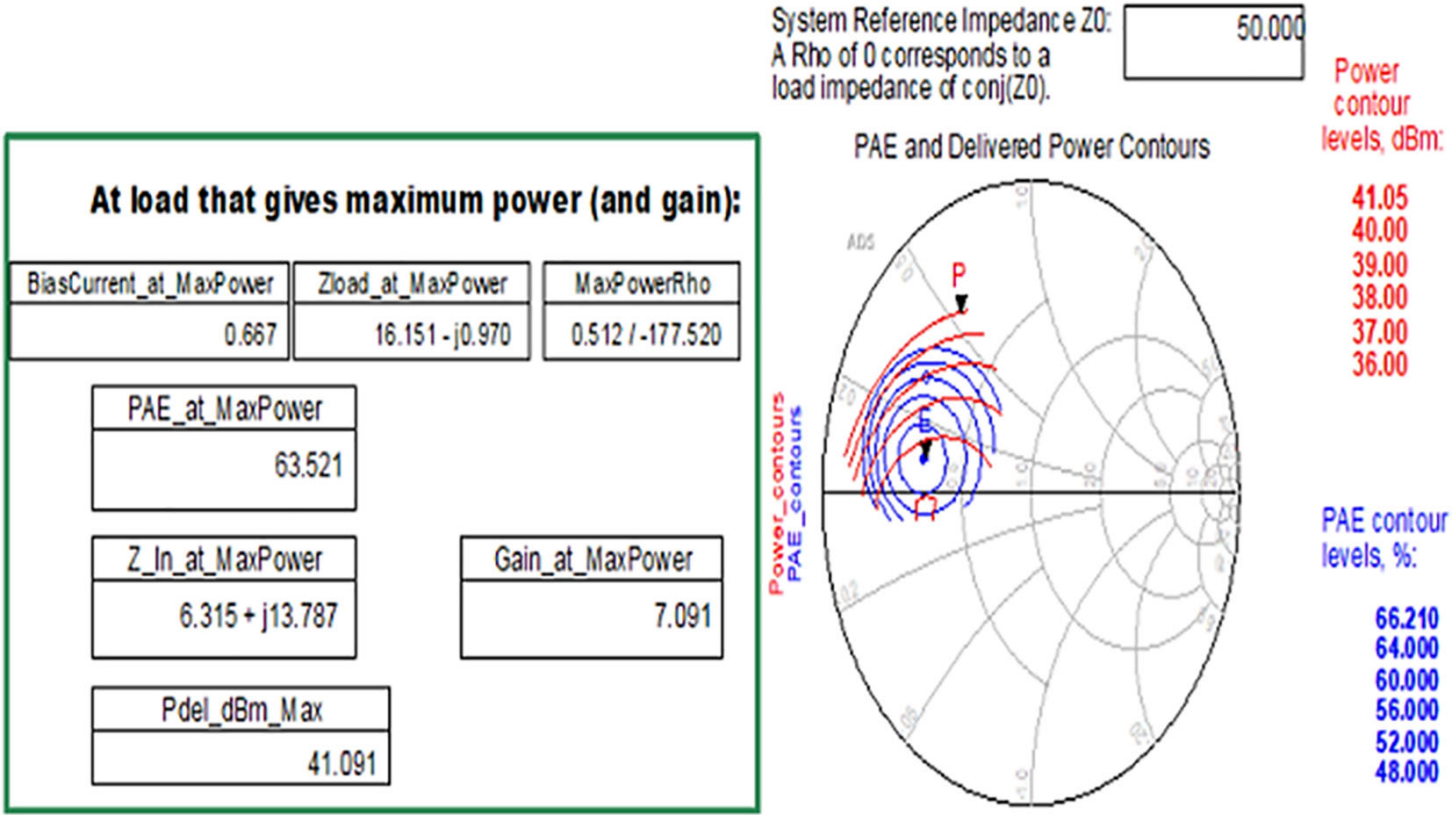
Optimum input and output impedances to obtain the maximum power delivered.

After checking the device’s stability, the optimum impedances required for the Class-J PA can be obtained by conducting load d pull simulations, as shown in Figure 7, with reference to the target source and load impedances that are obtained from load-line analysis, as explained in step 4 of the Methodology section. The optimum source/input impedance obtained from the load-pull analysis is 6.315+j*13.787, and the load/output impedance obtained is 16.151-j*0.970, corresponding to the maximum power delivered (Pdel_dBm_MAX), as shown in Figure 17.

**Figure 18.**
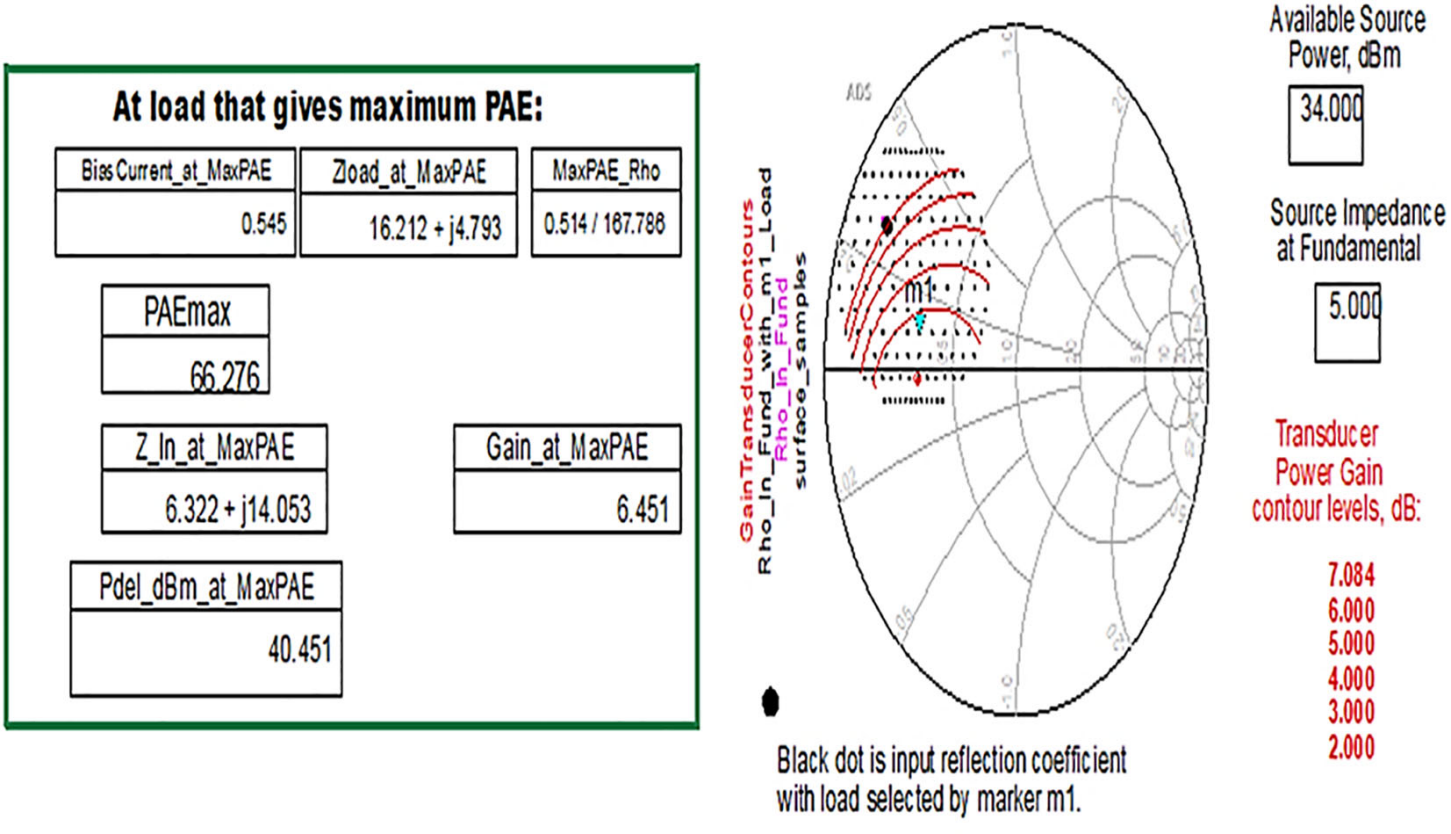
Optimum input and output impedances to obtain the maximum PAE.

Similarly, the optimum source/input impedance obtained is 6.322+j*14.053, and the load/output impedance obtained is 16.212+j*4.793, corresponding to the maximum PAE of 66%, as shown in Figure 18.

From the results of load-pull analysis, the optimum input and output impedances corresponding to the maximum PAE are chosen for the Class-J PA design and tabulated by comparison with the target source and load impedances obtained from load-line analysis-based mathematical design equations, as shown in Table 2.

**Table 2.** Comparison of input and output impedances obtained from load pull for the corresponding max PAE with load-line-based theoretical values.

Impedance	PAE_Max (%)	P _out_ (dBm)	Load-pull	Theory/load line
**Source/input**	66.276	40.451	6.3+j*14.1 Ω	5+j*0 Ω
**Load/output**	66.276	40.451	16.2+j*4.8 Ω	22.3+j*28.4 Ω

**Figure 19.**
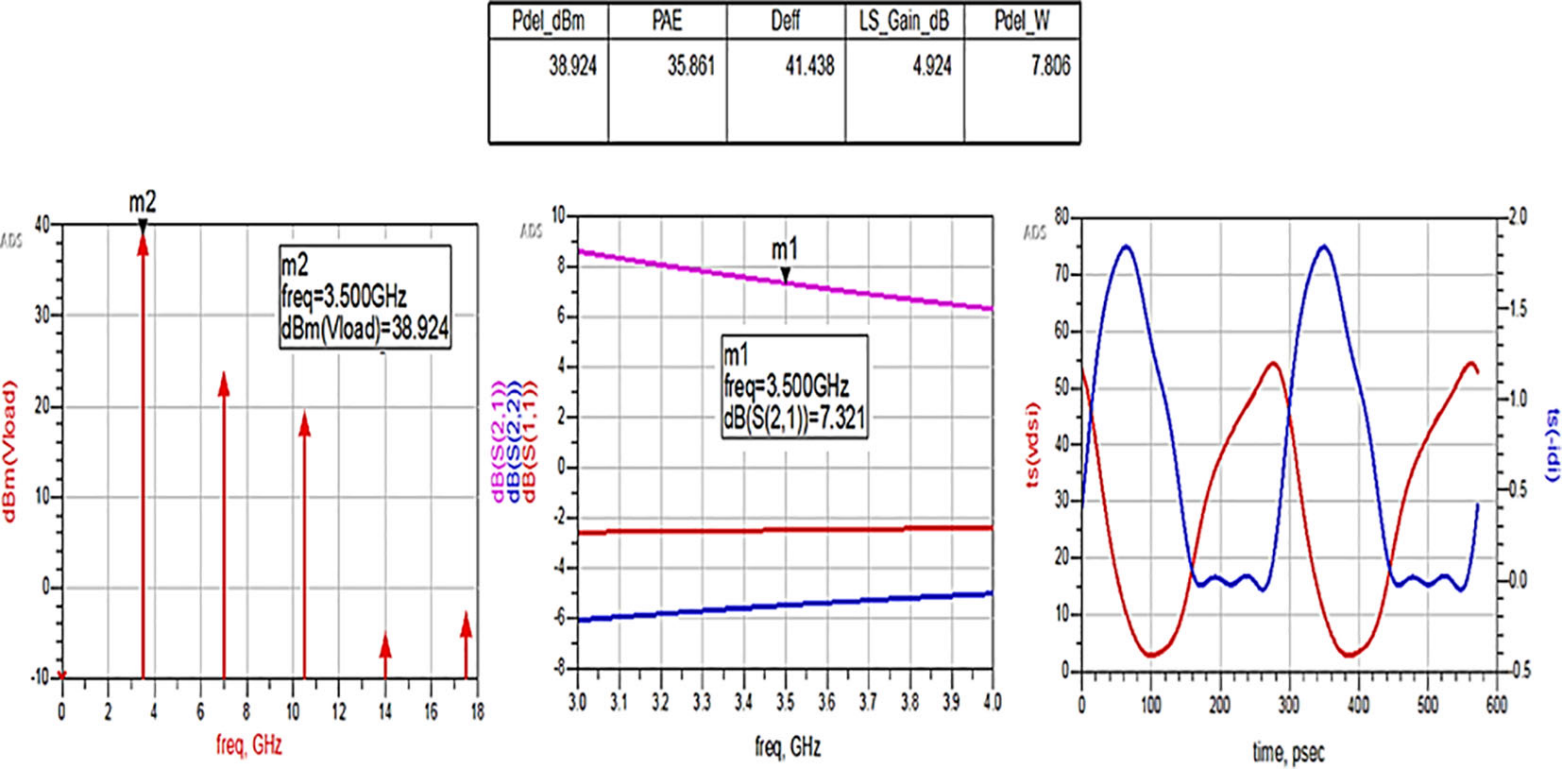
Performance parameters corresponding to a 50 Ω source and load terminations.

Before validating the optimum source and load impedances, the PA is terminated to a 50 Ω source and load terminals and the corresponding performance parameters, as shown in Figure 19.

Figure 19 shows that the performance parameters such as power delivered, large-signal and small-signal gains, D. E, and PAE are very low, as the CGH40010F GaN transistor does not terminate to optimum impedance values.

**Figure 20.**
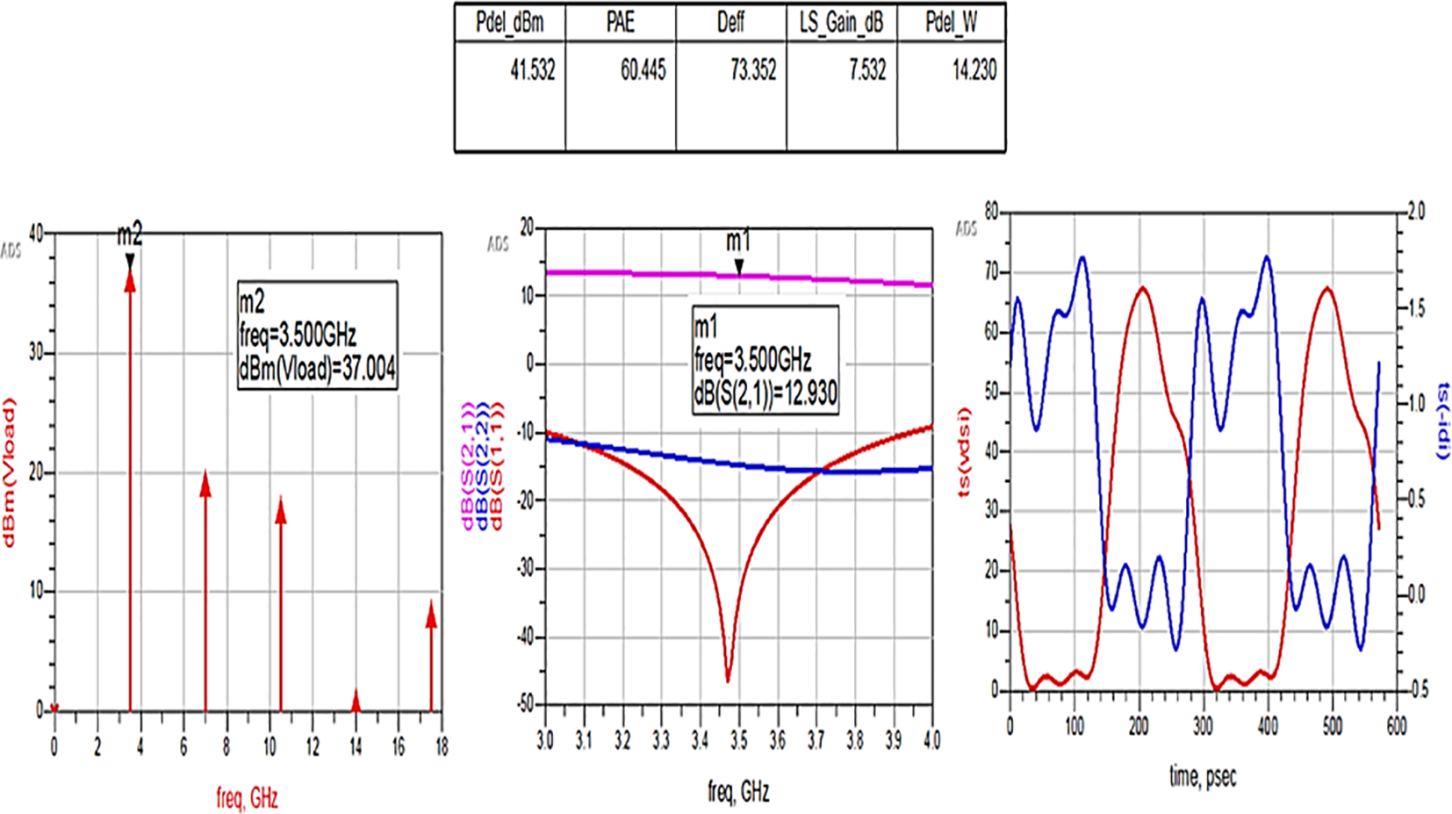
Performance parameters corresponding to the optimum source and load terminations without matching networks.

By terminating the transistor with the optimum impedance values obtained using load-pull simulations corresponding to the maximum PAE, harmonic balance S-parameter simulations are validated by running, as shown in Figure 8, and the respective performance parameters are shown in Figure 20.

From Figure 20, the performance parameters such as power delivered, large-signal and small-signal gains, drain efficiency and PAE are improved and nearly equal to the values obtained from load-pull simulations, as the input and output impedances of the CGH40010F GaN transistor are terminated to optimum impedance values. In addition, the reflection coefficients S (1,1) and S (2,2) are not as expected.

**Figure 21.**
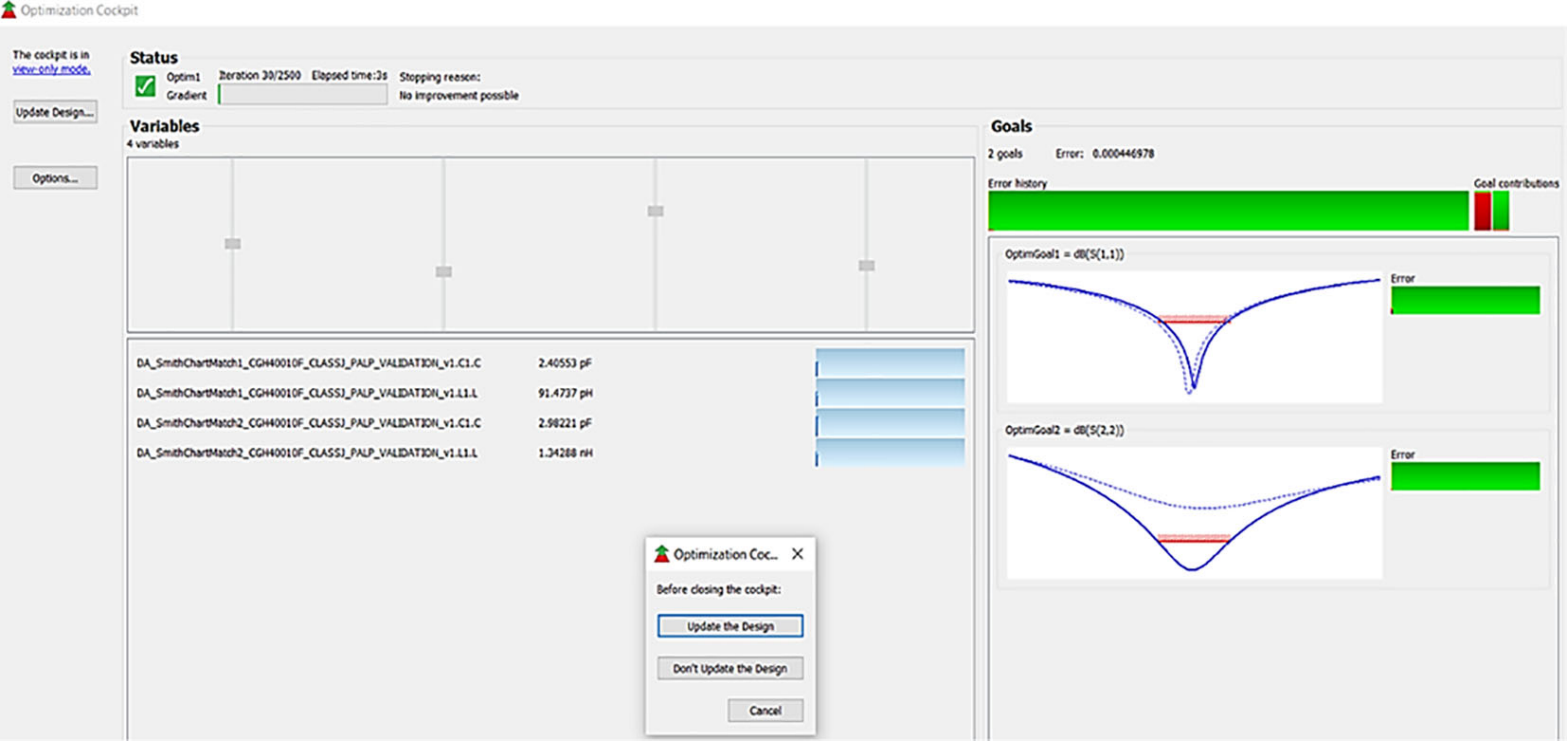
Optimization of input and output matching network elements.

After validation of optimum impedances obtained from load-pull analysis, the M. Ns are designed as explained in step 7 of the Methodology section and placed at the respective input and output terminals of the PA, as Smith chart components are shown in Figure 13. Finally, these matching network elements are optimized and updated using an optimization tool in ADS, as shown in Figure 21.

**Figure 22.**
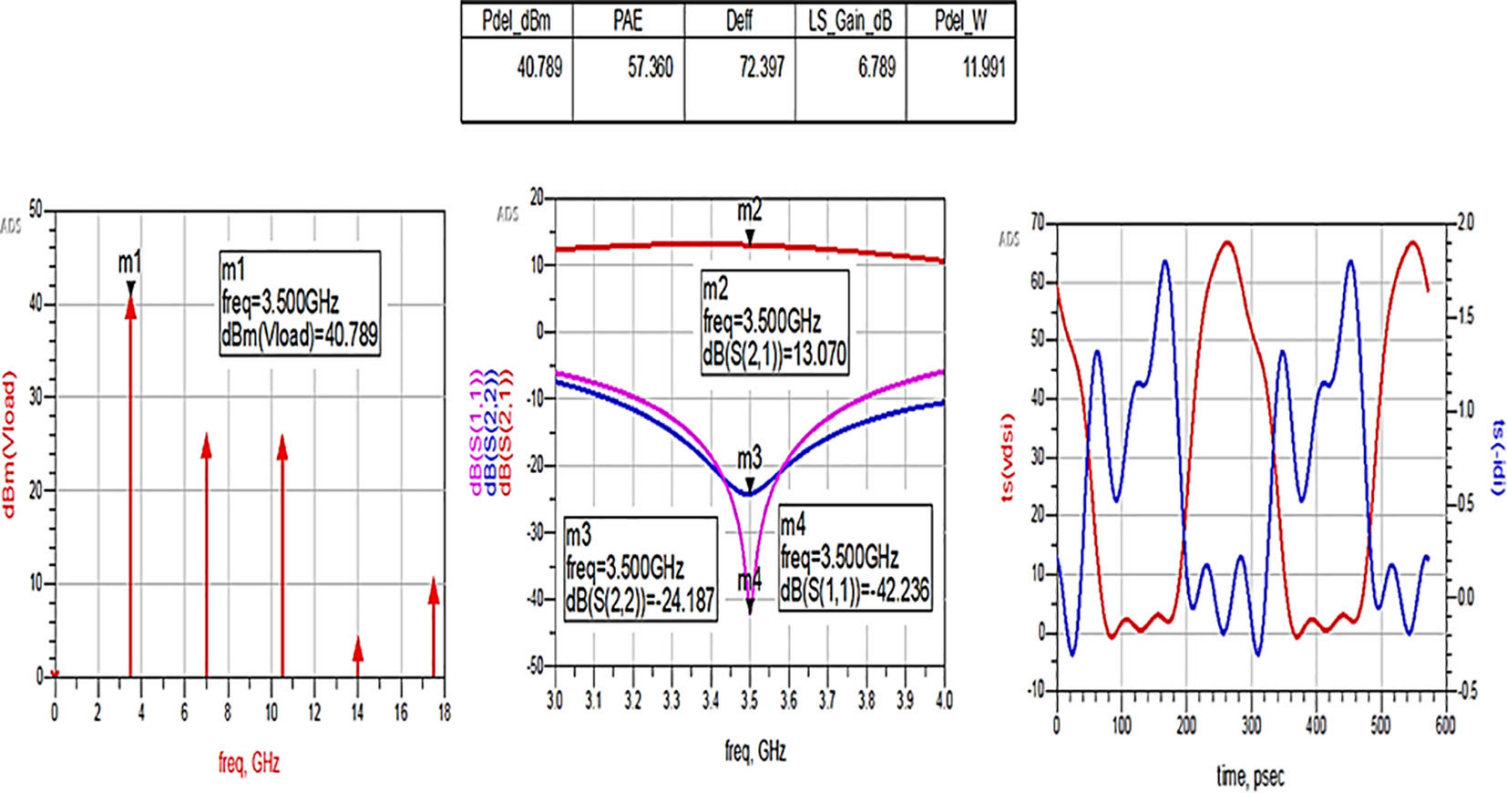
Performance parameters corresponding to the optimum source and load terminations using Smith chart utility-based matching networks.

After optimizing the performance parameters such as power delivered and large-signal and small-signal gains, PAE and D. E are shown in Figure 22. The reflection coefficients S (1,1) and S (2,2) are obtained as expected with the optimization of input and output matching networks.

**Figure 23.**
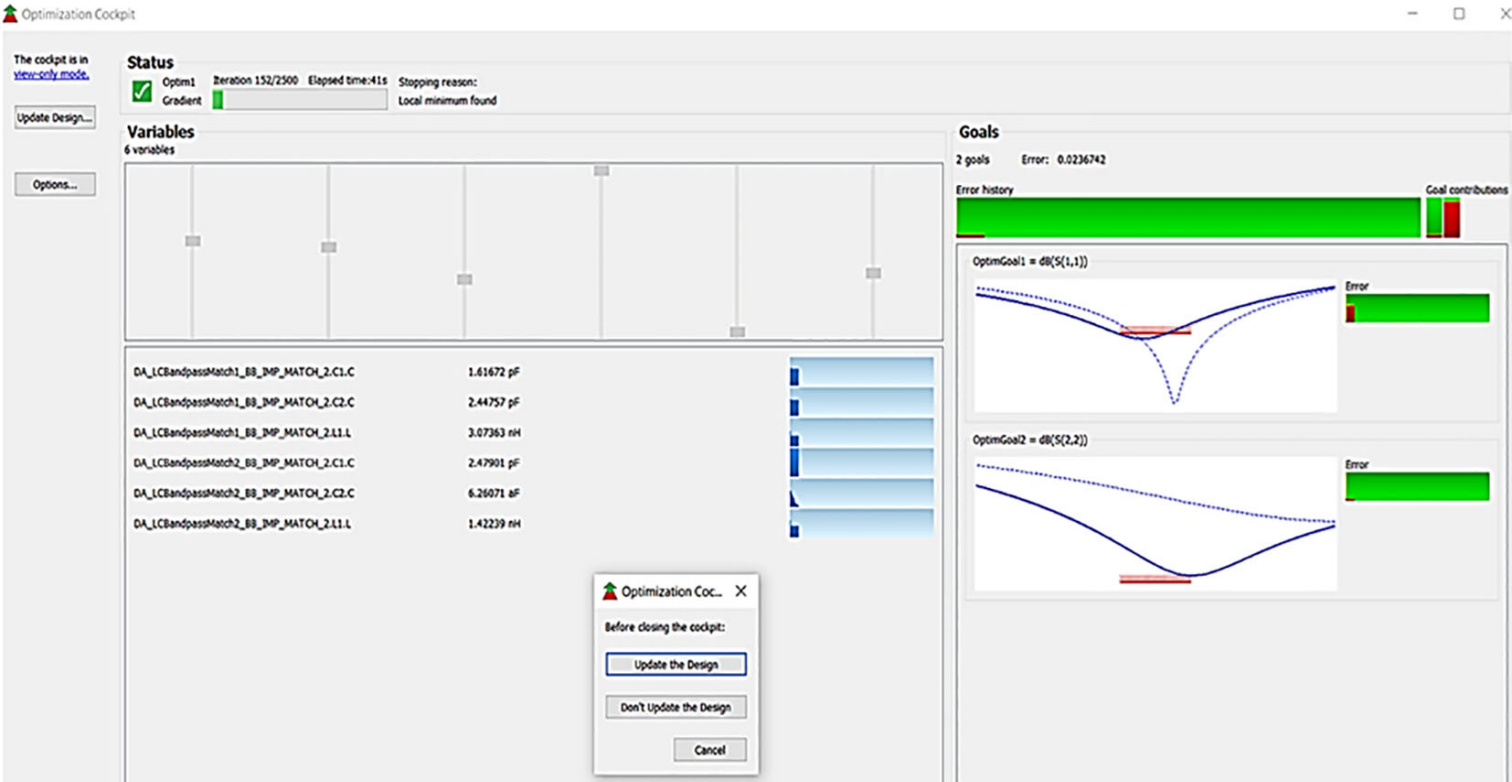
Optimization of input and output matching network elements.

The matching networks that are designed using L.C. bandpass match with the impedance matching utility, as explained in step 7 of the Methodology section, are placed at respective input and output terminals of the PA, as shown in Figure 14. Then, these matching network elements are optimized and updated using an optimization tool in ADS, as shown in Figure 23.

**Figure 24.**
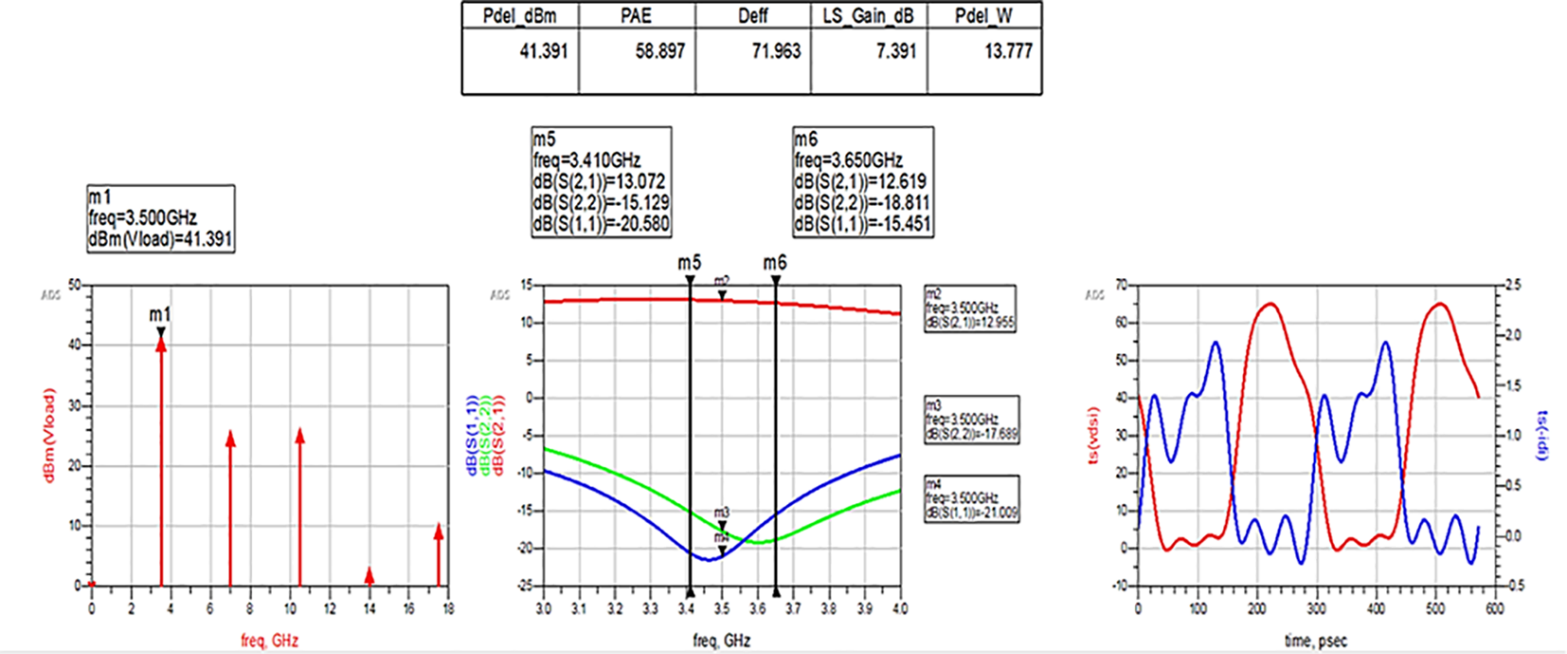
Performance parameters corresponding to the optimum source and load terminations using LC bandpass matching networks.

After optimizing the performance parameters such as power delivered and large-signal and small-signal gains, PAE and D. E are shown in Figure 24. The performance parameters are improved slightly with the optimization of the matching networks that are designed using the impedance matching utility.

**Figure 25.**
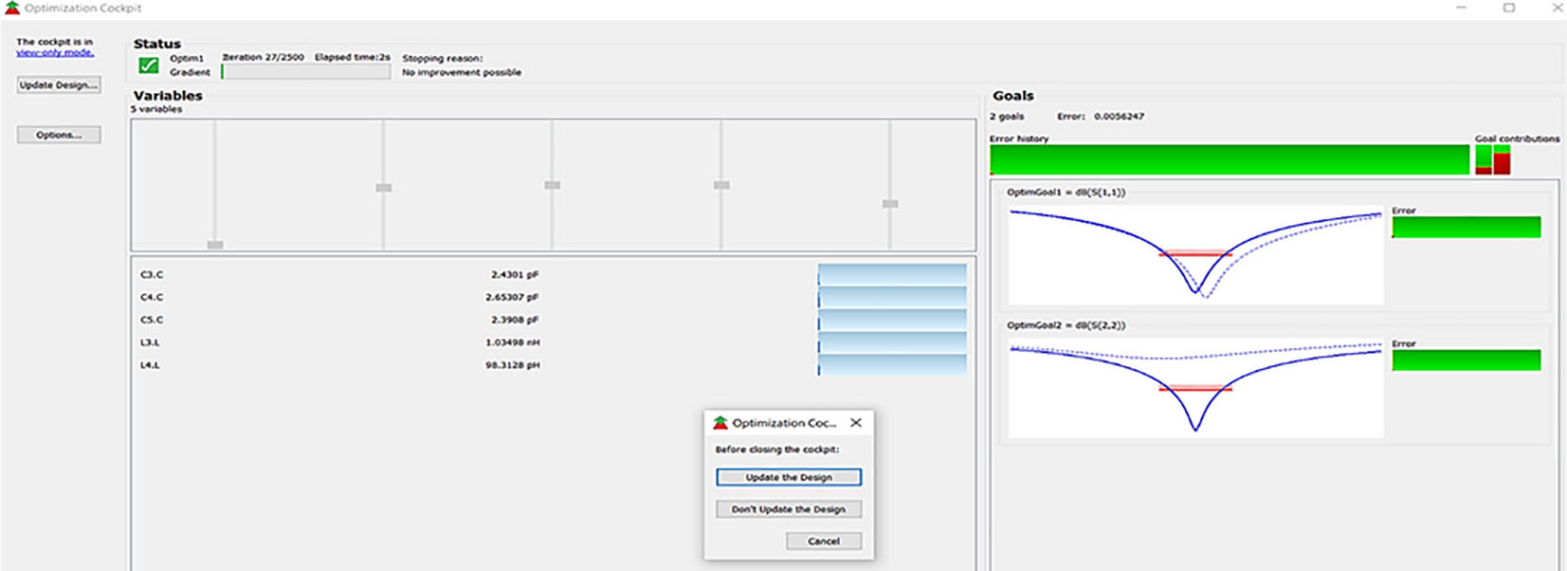
Optimization of input and output matching network elements.

The matching networks that are designed using basic L-type input and π-type output matching design equations, as explained in step 7 of the Methodology section, are placed at the input and output terminals of the PA, as shown in Figure 15, and then these matching network elements are optimized and updated using an optimization tool in ADS, as shown in Figure 25.

**Figure 26.**
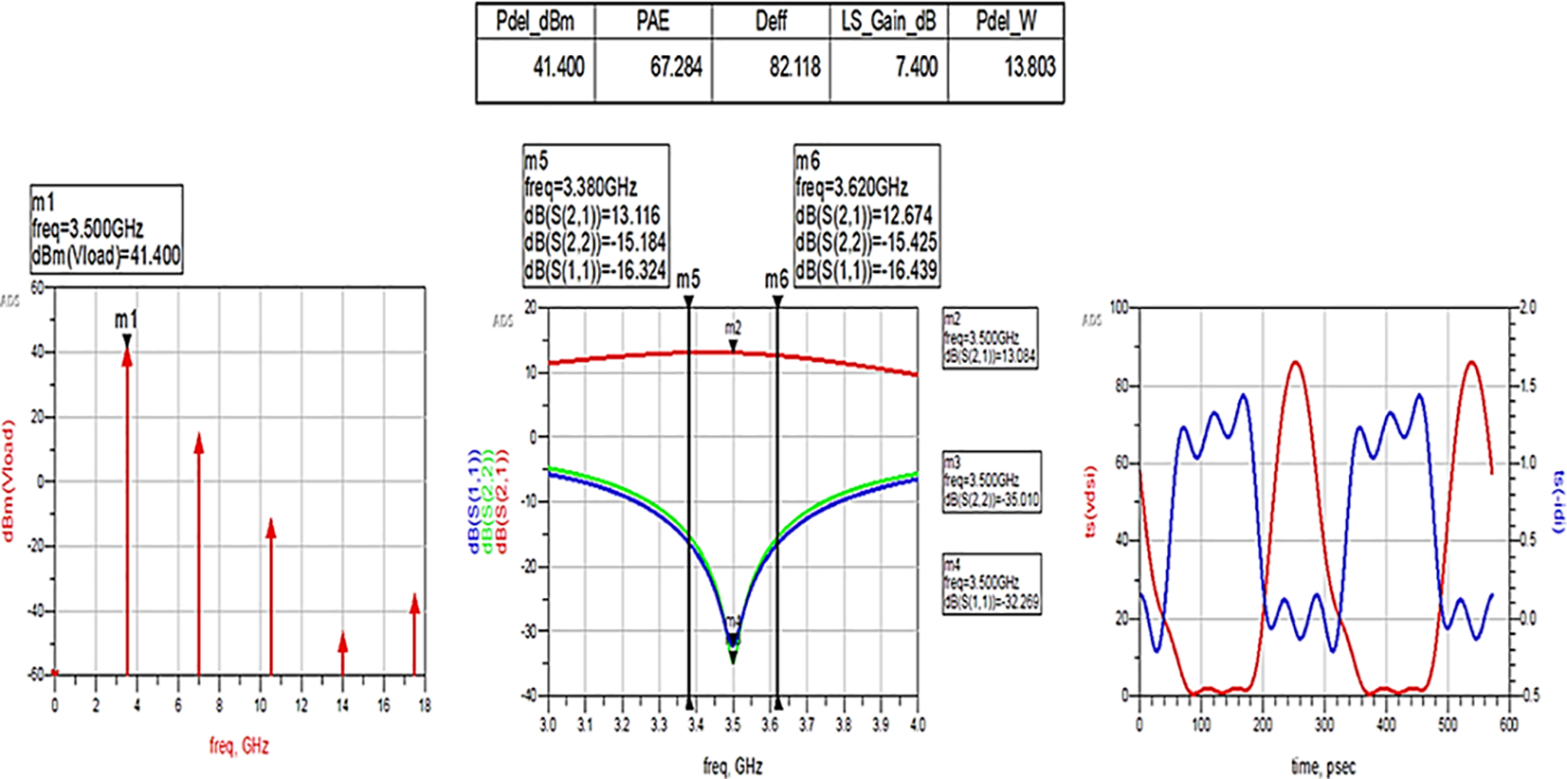
Performance parameters corresponding to the optimum source and load terminations using L-type input and π-type output matching networks.

After optimizing the performance parameters such as power delivered and large-signal and small-signal gains, PAE and D. E are shown in Figure 26. The performance parameters are obtained as expected with the optimization of input and output matching networks.

All the numerical values of performance parameters such as power delivered (Pdel_W, Pdel_dBm_MAX) in watts and dBm, large-signal gain (LS_Gain_dB), PAE and D. E (Deff) that are shown in Figures 19, 20, 22, 24, and 26, corresponding to without and with three matching topologies, are computed with many equations using the “MeasEqn” of the harmonic balance HB simulator. The load voltage is plotted as the spectrum in dBm, and intrinsic voltages/currents are plotted as time-domain signals using an (HB) simulation controller in ADS. The desired half-rectified intrinsic drain voltage and currents with small overlap that represent the Class-J mode of operation were obtained, and reflection coefficients S (1,1) and S (2,2) > -10 dB over a frequency range from (3.3–3.7) GHz were observed from Figure 26. The comparison of the performance parameters of the Class-J PA with and without matching networks obtained for different source and load terminations is shown in Table 3.

**Table 3.** Performance comparison of the proposed Class-J PA with and without matching networks (M.Ns).

Mode of source & Load termination	50 Ω without M.N	Optimum ZS and ZL without M.N	50 Ω with Smith chart utility-based M.N	50 Ω with impedance match utility-based M.N	50 Ω with L-type I/P and π-type O/P M.N
Feature	Class-J	Class-J	Class-J	Class-J	Class-J
Freq [GHz]	3.5	3.5	3.5	3.5	3.5
Vsupply [V]	28	28	28	28	28
LS_Gain [dB]	5	7	7	7.4	7.4
SS_Gain [dB]	7	12.9	13	12.95	13.1
Pout [dBm]	39	41.5	41	41.4	41.4
max PAE [%]	36	60	57	59	67
max DE [%]	41	73	72	72	82
BW [GHz]	3–4	3–4	3–4	3–4	3–4

The above comparison table shows that among the 3 methods used for matching networks, the Class-J PA with L-type input and π-type output matching networks exhibits expected performance parameters that are obtained from load-pull simulations (i.e., 66% of PAE and 40 dBm of max power output), as shown in Figure 18.

**Figure 27.**
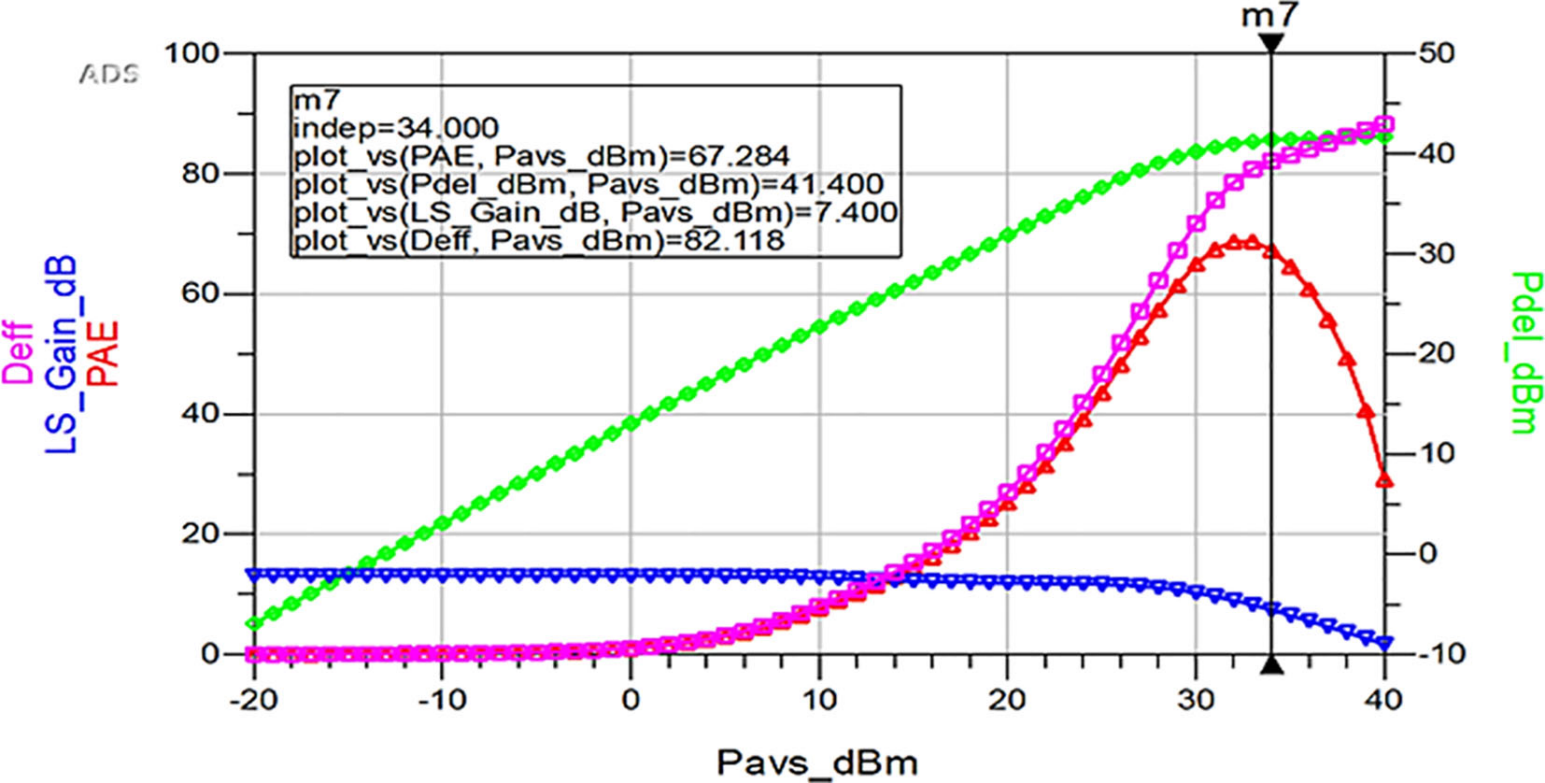
Performance parameters of the Class-J PA w.r.t Pavs_dBm sweep.

To validate the variation in the performance parameters with respect to the input power sweep, HB simulations were performed on the proposed Class-J PA by taking the available source power (Pavs_dBm) as the sweeping parameter. Figure 27 shows the simulation results of important performance parameters such as drain efficiency, PAE, large-signal gain, and power delivered corresponding to the available source power (Pavs_dBm) sweep. These results demonstrate that, a maximum power output of 41.4 dBm with a power gain of approximately 7.4 dB, max D. E of 82%, and max PAE of 67% for the corresponding available source power of 34 dBm with a 28 V power supply into a 50 Ω load.

**Figure 28.**
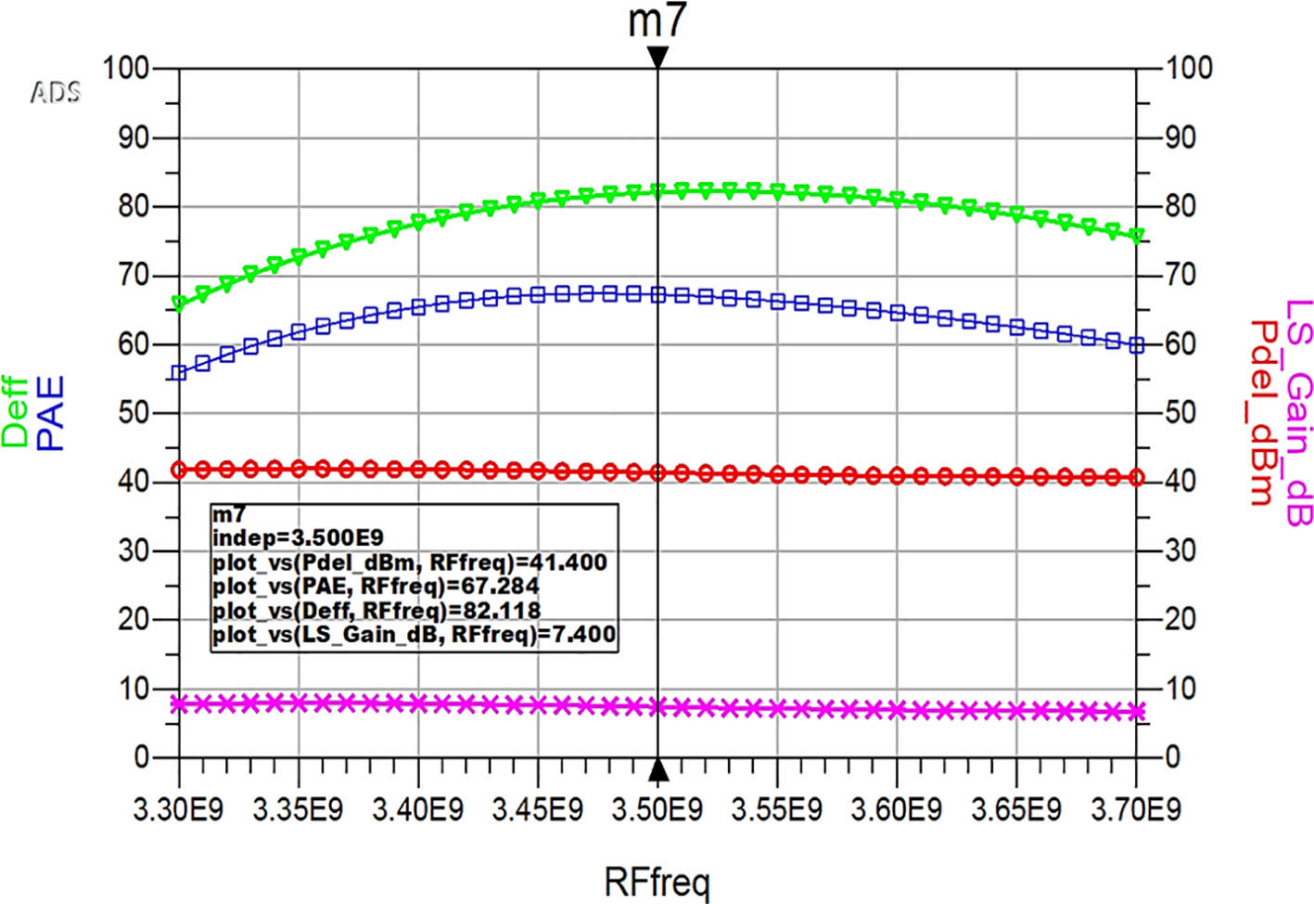
Performance parameters of the Class-J PA w.r.t R.F. frequency.

Similarly, HB simulations were performed to validate the performance parameter variation of the proposed Class-J PA with L-type input and π-type output matching networks with respect to the input RF frequency, and the corresponding results are displayed in Figure 28. These results reveal that a power output above 40 dBm with a power gain of approximately 7 dB over a bandwidth of approximately 400 MHz (i.e., 3.3 GHz to 3.7 GHz) and max PAE and D. E of 67% and 82%, respectively, are obtained at a 3.5 GHz center frequency.

**Figure 29.**
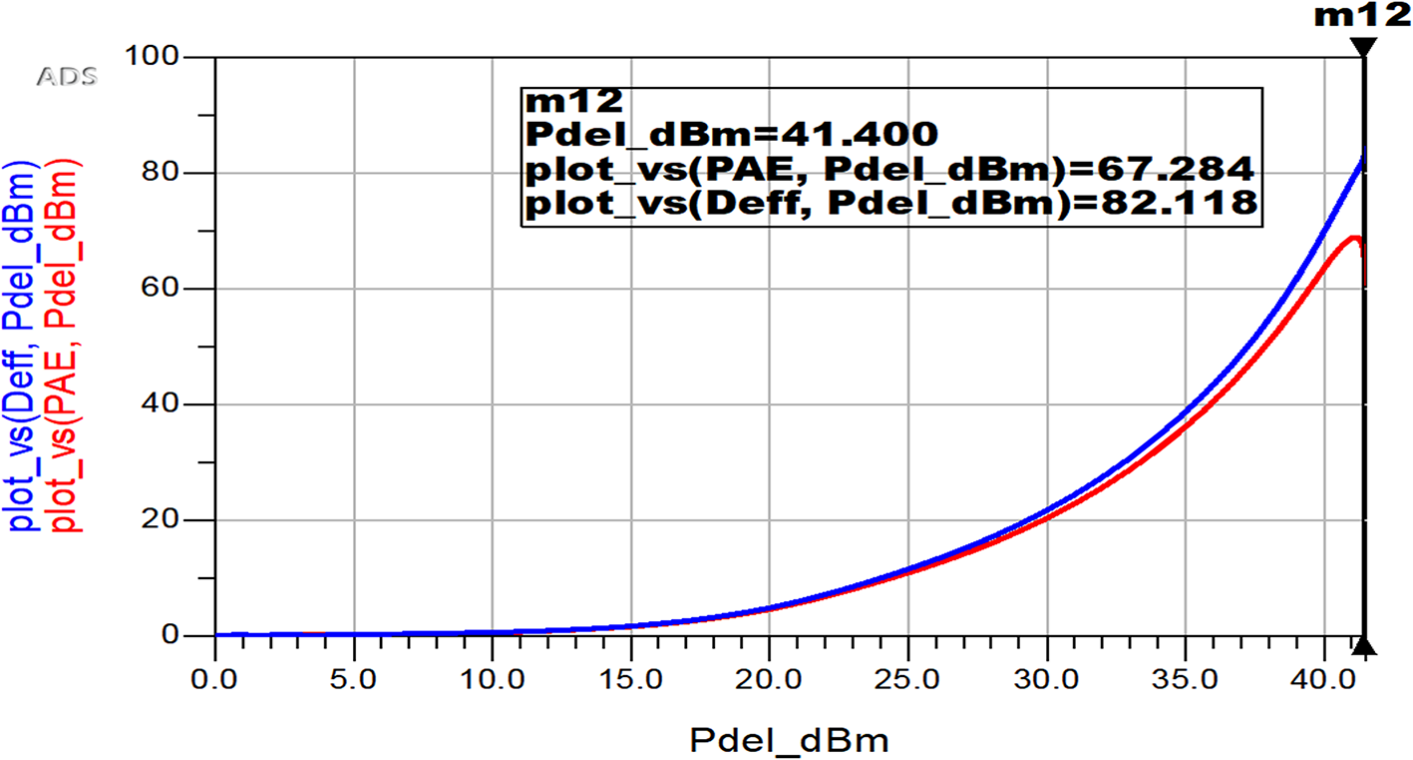
Power efficiency (Deff, PAE) Vs power output (Pdel_dBm).

The variation of proposed Class-J PA’s D. E and PAE corresponding to its output power is illustrated in Figure 29.

It is observed that the PAE and Drain efficiency of the proposed Class-J PA are very low at low power levels and typically increase as the PA reaches higher output power levels and maintains high efficiency even at elevated power levels due to the efficient switching characteristics of GaN transistors and the design of Class-J mode, which helps minimize power dissipation.

The linearity of the proposed Class-J PA is another crucial parameter to evaluate its performance. In general, it can be evaluated using metrics like P1dB/P3dB (1 dB/3 dB compression point), third-order intercept point, AM-PM distortion, adjacent channel power ratio, etc. depending on its input signal and transistor technology used.

In this work, the proposed Class-J PA’s linearity is evaluated using a 3 dB compression point. Because it is designed using a GaN transistor as the main active device and fed by a continuous wave (CW)/1-tone signal as its RF input. The 3 dB compression point is defined as the point at which the gain of the PA begins to compress due to nonlinearity, and the output power no longer increases linearly with the input power.

**Figure 30.**
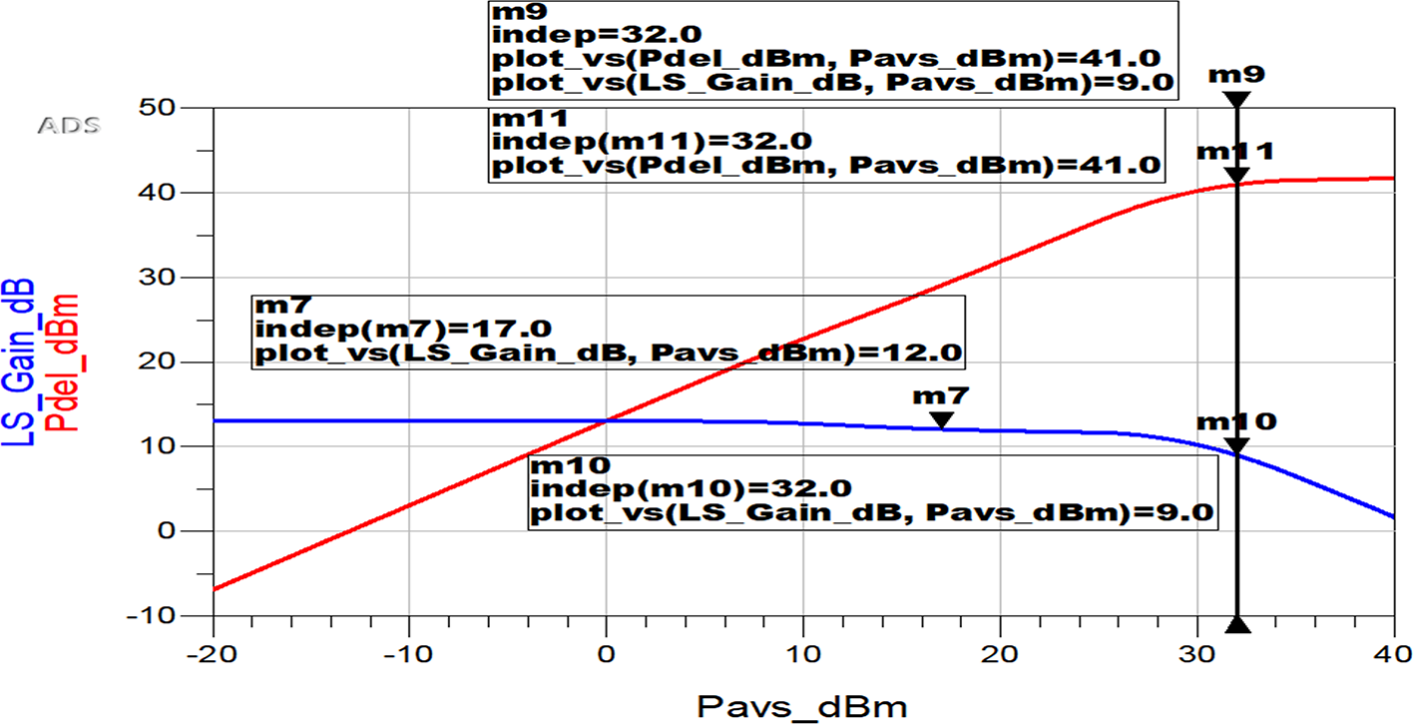
Linearity of the Class-J PA using 3 dB compression point.

According to the simulation findings, the gain of the proposed PA is compressed by 3 dB (from 12 dB to 9 dB), as shown in Figure 30. At this 3 dB compression point, the Pout value is 41 dBm, which is nearly as high as the saturated power (Psat) value (i.e., 41.4 dB), revealing the proposed Class-J PA’s linearity.

The proposed Class-J PA is designed and optimized for achieving efficiency. However, it offers good noise performance as it uses a GaN transistor, which generally exhibits low intrinsic noise characteristics compared to other semiconductor technologies like GaAs. This is due to GaN’s high electron mobility and low on-resistance, which contribute to better overall noise performance. In addition, the minimal impact of noise on the signal integrity is ensured by proper impedance M. Ns design and is validated through the linearity of the proposed PA.

## Discussion

Finally, the improved performance parameters of the proposed Class-J PA with L-type input and π-type output matching networks are compared with similar Class-J PAs in the literature. ^30^^-^^34^ As these PAs were designed at different frequencies and technologies, it is difficult to compare their performances. However, it can be noted that the Class-J PA design is proposed with a compact transmission line-based output matching network (OMN) in ^30^ that uses the same transistor technology (GaN) of this work to obtain broadband and highly efficient amplification and achieves a maximum drain efficiency of 75%. Although our proposed PA is designed at different frequencies, its bandwidth is the same as that of this PA, and its transmission line-based M. Ns use a large chip area compared to the M. Ns of our work. A microwave Class-J PA for Wi-Fi IEEE802.11a Bluetooth applications designed with a methodology similar to that of this work in the same ADS EDA tool is presented in, ^31^ which uses a GaAs transistor as an active device but achieves a power output of 21 dBm and D. E of 69%. A fully integrated Class-J PA designed at 5 GHz for WLAN 802.11ax applications was presented in ^32^ that uses GaN on SiC technology with the same V dd=28 V to provide a maximum PAE of 55% and output power of 38 dBm, and it was mentioned that the performance of this PA can be enhanced further by employing DPD. However, the gain of this PA is high compared to our work, as it is designed as a multistage PA. A Class-J PA designed for X-Band is presented in. ^33^ It uses the active load modulation technique, facilitates the PA's integrated implementation by eliminating the doubler and filter networks of conventional Class-J2 PAs, and achieves a drain efficiency of 71% and a PAE of 50%. However, the broad B.W. is not achieved because of harmonic tuners, and the auxiliary network used for phase shifting may need additional circuitry and space. A Class-J PA design with a novel direct M. N synthesis technique for broadband operation is presented in. ^34^ The PAE and output power (dBm) are almost the same at the frequency (3.5 GHz) of our proposed work. Although the synthesis technique is novel, the transmission line-based MNs may occupy a large chip area. In addition, an integrated Class-J PA using CMOS technology is presented in, ^22^ in which the effect of knee voltage is considered for deriving modified design equations. However, the staked FET must be used for implementation because of the CMOS PA’s low breakdown voltage, whereas our work uses a GAN device with a high breakdown voltage.

The performance comparison discussed thus far is summarized in Table 4. The PAE and DE of this work are better, and the BW is comparatively lower than those of other works shown in Table 4. Because, in this work initially the proposed class J PA’s M. Ns are designed with a smaller number of lumped elements to obtain high efficiency, which leads to a bandwidth of 400 MHz with a centre frequency of 3.5 GHz. Although the BW obtained is comparatively lower than the existing Class-J PA designs in Table 4, it will be sufficient to implement the PA in emerging 5G wireless networks of smart metering/AMI applications. However, its BW can be enhanced further by replacing the passive lumped inductor with an AI through its tunability. We report that the proposed Class-J PA design of this work is not relatively efficient than other switching mode PAs. However, it has achieved a high efficiency without sacrificing the linearity over the desired BW compared with the existing author’s Class-J PA designs shown in Table 4.

**Table 4.** Performance Comparison of Class-J PAs.

REF	This work	30	31	32	33
Technology	GaN	GaN	GaAs	GaN	GaAs
Feature	Class-J	Class-J	Class-J	Class-J	Class-J
Freq. [GHz]	3.5	2	2.4	5	10
Pout [dBm]	41.4	42	21	37.7	29
max PAE [%]	67	66	-	54.6	50
max DE [%]	82	75	69	-	71
BW [GHz]	3.3–3.7	1.8–2.2	-	4.9–5.9	9.1–10.8

In this work, the key factors such as efficiency, Pout, BW, and linearity of PA are considered to check its suitability for implementation in 5G wireless networks of smart metering applications. However, for its real-world deployment in 5G smart-meter wireless networks, the proposed PA’s chip area can be estimated to check its integration feasibility through the active element-based designs such as Active Inductor (AI) for its OMN.

## The main contribution/novelty

The main contribution/novelty of this work involves designing of proposed Class-J PA’s M. Ns corresponding to 3.5 GHz centre frequency (sub 6 GHz 5G frequency) in 3 different ways using Smith chart utility, impedance match utility, and basic L-type and π-type impedance matching network design equations with an idea to replace the passive lumped inductor in its OMN with an AI, to achieve desired performance in terms of efficiency over enhanced B. W along with the linearity for making it suitable for emerging 5G wireless networks in smart metering or AMI applications.

## Conclusions

A 3.5 GHz Class-J PA design with lumped element-based input and output matching networks that are suitable for 5G smart meter/grid applications is presented in this paper. The proposed Class-J PA design methodology is demonstrated in a stepwise manner. It is observed that with a small overlap between the intrinsic half-wave rectified current and voltage waveforms at the drain, this Class-J mode PA can be as linear as Class-B or AB modes because of its non-switching mode of operation. From the simulation results, we conclude that the proposed Class-J PA obtains a maximum drain efficiency of 82%, which is better than similar Class-J PAs reported in the literature. A PAE of 67% with a 13 dB small-signal gain at 3.5 GHz and an output power of 40 dBm (41.4 dBm peak) with a power gain of approximately 7 dB over a bandwidth of approximately 400 MHz (i.e., 3.3 GHz to 3.7 GHz) are achieved with no harmonic traps, unlike in Class-B mode PAs, which makes this Class-J PA superior to other PA modes and more appealing for emerging wireless communication networks used for AMI of smart meters in 5G smart grids. The proposed Class-J PA contributes to the overall functionality and performance of 5G smart metering applications by extending communication range through its high-power efficiency, increasing data transmission speed with wider BW, and improving reliability with reduced signal distortion. However, the PAE and BW of the proposed Class-J PA can still be improved by fine-tuning the designed matching networks and there is a scope for realizing the proposed Class-J PA with active element-based design to reduce its chip area and make it feasible for integration. This work is in progress to achieve the desired specifications of 5G wireless networks of smart grid applications.

## Data availability

No data are associated with this article.

## Author contributions

Mr. Nagisetty Sridhar-original drafting of manuscript preparation, Dr Chinnaiyan Senthilpari-validation and Supervision, Dr. Mardeni R-cosupervision. All the authors agreed to the final version of this manuscript.
