# Supplementary material for: An enhanced broadband class-J mode power amplifier for 5G smart meter applications
Source: F1000Res. 2024 Sep 12;10:1099. Originally published 2021 Oct 29. [Version 2] doi: 10.12688/f1000research.73498.2 (PMC11523559; doi:10.12688/f1000research.73498.2)
Supplement: Peer review history

# Reviewer report: Reviewer response for version 2

**DOI:** 10.5256/f1000research.171375.r323101

**Published:** 2024-10-28

**Authors:** Tahesin Samira Delwar

**Affiliations:** 1 Pukyong National University, Busan, South Korea

The author has addressed all comments well. The revised version is recommended to be accepted.

Is the work clearly and accurately presented and does it cite the current literature?

Partly

If applicable, is the statistical analysis and its interpretation appropriate?

Not applicable

Are all the source data underlying the results available to ensure full reproducibility?

Partly

Is the study design appropriate and is the work technically sound?

Partly

Are the conclusions drawn adequately supported by the results?

Partly

Are sufficient details of methods and analysis provided to allow replication by others?

Partly

Reviewer Expertise:

My current research interests are RF circuit design, high-efficiency CMOS power amplifiers, Optical wireless communication systems and visible-light communications.

I confirm that I have read this submission and believe that I have an appropriate level of expertise to confirm that it is of an acceptable scientific standard.

# Reviewer report: Reviewer response for version 1

**DOI:** 10.5256/f1000research.77153.r229222

**Published:** 2024-08-02

**Authors:** Tahesin Samira Delwar

**Affiliations:** 1 Pukyong National University, Busan, South Korea

How does the proposed amplifier manage harmonic distortion across the desired frequency bands? Providing a thorough harmonic analysis, especially in the context of 5G frequency ranges, would be insightful.

 What is the power efficiency of the enhanced Class-J mode power amplifier, particularly at varying output power levels?

 What is the noise performance of the amplifier, and how does it impact the signal integrity in 5G smart meter applications? Discussing noise considerations is essential for applications demanding high-quality signal transmission.

 What considerations should be taken into account for the real-world deployment of the proposed amplifier in 5G smart-meter networks? Discussing practical considerations, such as cost, power consumption, and scalability, would be valuable for potential adopters.

 How closely do the experimental results align with the simulation findings?

 Improve the figure quality.

 Ensure that the references are up-to-date and include recent contributions in the field of power amplifiers for 5G applications. Add some recent journal reference papers instead of conference papers.

 Make a subsection of the main contribution and related literature of the research.

 Highlight the novelty of the proposed circuit.

 Presentation skills are very poor. Improve the presentation skills.

 Add the future research scope.

 Linearity analysis is missing.

 Considering the practical deployment of 5G smart meters, were there any integration challenges encountered with the proposed amplifier?

 How does the enhanced broadband Class-J mode power amplifier contribute to the overall functionality and performance of 5G smart meters? Discussing the impact on communication range, reliability, and data transmission speed would be valuable.

Is the work clearly and accurately presented and does it cite the current literature?

Partly

If applicable, is the statistical analysis and its interpretation appropriate?

Not applicable

Are all the source data underlying the results available to ensure full reproducibility?

Partly

Is the study design appropriate and is the work technically sound?

Partly

Are the conclusions drawn adequately supported by the results?

Partly

Are sufficient details of methods and analysis provided to allow replication by others?

Partly

Reviewer Expertise:

My current research interests are RF circuit design, high-efficiency CMOS power amplifiers, Optical wireless communication systems and visible-light communications.

I confirm that I have read this submission and believe that I have an appropriate level of expertise to confirm that it is of an acceptable scientific standard, however I have significant reservations, as outlined above.

# Response

**Published:** 2024-09-03

**Authors:** SRIDHAR NAGISETTY

Dear Reviewer

 I would like to thank you for your valuable comments. I have amended the manuscript as per your comments and the corresponding point-to-point response is given below:

**Reviewer Comment-1: **How does the proposed amplifier manage harmonic distortion across the desired frequency bands? Providing a thorough harmonic analysis, especially in the context of 5G frequency ranges, would be insightful.

**Response**: Thank you, Prof., The proposed class-J PA is designed and optimized to minimize harmonic distortion and enhance the PAE and Drain Efficiency across the desired bandwidth using load pull techniques and careful design of its OMN, as per your suggestion, it is explained just **below Figure 15 **before the **Results** section.

**Reviewer Comment-2: **What is the power efficiency of the enhanced Class-J mode power amplifier, particularly at varying output power levels?

**Response: **Thank you, Prof., As per your suggestion, the variation of PAE and Drain efficiency of the proposed class-J PA corresponding to the output power levels is discussed by including **Figure 29** just **before the discussion section** of the manuscript.

**Reviewer Comment-3: **What is the noise performance of the amplifier, and how does it impact the signal integrity in 5G smart meter applications? Discussing noise considerations is essential for applications demanding high-quality signal transmission.

**Response: **The proposed class-J PA offers good noise performance as it uses a GaN device as the main transistor. As per your suggestion, noise performance proposed class-J PA of and its impact on signal integrity is explained **below Figure 30, just before the discussion section** of the manuscript.

**Reviewer Comment-4: **What considerations should be taken into account for the real-world deployment of the proposed amplifier in 5G smart-meter networks? Discussing practical considerations, such as cost, power consumption, and scalability, would be valuable for potential adopters.

**Response: **The key factors considered while deploying the proposed class-J PA in 5G wireless networks of smart metering applications are explained in the discussion section **after Table 4.**

**Reviewer Comment-5: **How closely do the experimental results align with the simulation findings?

**Response**: Thank you, Prof., In this work, the proposed class-J PA is designed with an idea to replace the passive inductor in the OMN with an Active Inductor. Therefore, in this paper, only simulation results of proposed class-J PA that is suitable for emerging 5G wireless networks in smart metering/AMI applications, with 3-types of lumped element-based M.Ns designs as the main contribution, are presented.

**Reviewer Comment-6: **Improve the figure quality.

**Response: **Thank you, Prof. As per your suggestion, along with the new **Figures 29 and 30**, all other Figures are resubmitted as separate **300 dpi TIFF format files** as per the author's guidelines.

**Reviewer Comment-7: **Ensure that the references are up-to-date and include recent contributions in the field of power amplifiers for 5G applications. Add some recent journal reference papers instead of conference papers.

**Response**: Thank you, Prof., As per your suggestion, some of the conference papers are replaced with recent journal papers in the **REFERENCES **section of the manuscript.

**Reviewer Comment-8: **Make a subsection of the main contribution and related literature of the research. Highlight the novelty of the proposed circuit. Presentation skills are very poor. Improve the presentation skills.

**Response**: Thank you, Prof., As per your suggestion, the main contribution/ novelty of the proposed class-J PA is included as a **subsection** **before the conclusion section** in the manuscript. The presentation of paper has improved while addressing the reviewer comments.

**Reviewer Comment-9: **Add the future research scope

**Response**: Thank you, Prof., As per your suggestion The future scope of this research work is included in the **conclusion section**.

**Reviewer Comment-10: **Linearity analysis is missing.

**Response**: Thank you, Prof., As per your suggestion, the linearity Analysis of the proposed class-J PA using a 3 dB compression point is discussed by including **Figure 30 before the discussion section** of the manuscript.

**Reviewer Comment-11: **Considering the practical deployment of 5G smart meters, were there any integration challenges encountered with the proposed amplifier?

**Response**: Thank you, Prof., In this work the actual idea is to replace the passive inductor in the OMN of the proposed class-J PA with an Active Inductor. Therefore, in this paper, only simulation results of proposed class-J PA that is suitable for emerging 5G wireless networks in smart metering/AMI applications, with 3-types of lumped element-based M.Ns designs as the main contribution are presented.

**Reviewer Comment-12: **How does the enhanced broadband Class-J mode power amplifier contribute to the overall functionality and performance of 5G smart meters? Discussing the impact on communication range, reliability, and data transmission speed would be valuable

**Response**: The proposed class-J PA contributes to the overall functionality and performance of 5G smart metering applications by extending communication range through high power efficiency, increasing data transmission speed with wider bandwidth, and improving reliability with reduced signal distortion. This is included in the **conclusion section**.

# Reviewer report: Reviewer response for version 1

**DOI:** 10.5256/f1000research.77153.r163741

**Published:** 2023-11-02

**Authors:** Anumuthu Priya, Vanmathi M

**Affiliations:** 1 Department of Electronics and Communication Engineering, B.S. Abdur Rahman Crescent Institute of Science and Technology, Chennai, Tamil Nadu, India; 2 ECE, B S Abdur Rahman Crescent Institute of Science & Technology, Chennai, Tamil Nadu, India

This manuscript details the design of class- J power amplifier for 5G smart grid’s advanced metering infrastructure applications.

 Here are few comments from my side:

 1. The article is well written and the results are relatively interesting which I believe that the manuscript can provide some good insights for a future decision regarding 5G smart grid.

 2. However, as the class-J PA is well established, the authors should explain the novelty/contribution in the proposed class-J PA design.

 3. In Figure 6 authors are suggested to explain clearly how the phase shift and voltage boost are obtained by moving Alpha factor (α) from 0 to 1, in the relevant page?

 4. I would suggest the author to explain the reason for designing the matching networks of proposed PA by considering its optimum impedances corresponding to PAE rather than output Power.

 5. How the second harmonic condition of class-J mode operation is satisfied?

 6. Although the PAE and D.E of proposed PA are good its B.W is relatively low as per the comparison table, it is suggested to give some justification for obtained B.W of the proposed PA.

 7.Table 4 gives the performance comparison of class -J PA. Can the authors report this class -J pa is relatively efficient than other PA types also.

Is the work clearly and accurately presented and does it cite the current literature?

Yes

If applicable, is the statistical analysis and its interpretation appropriate?

Not applicable

Are all the source data underlying the results available to ensure full reproducibility?

No source data required

Is the study design appropriate and is the work technically sound?

Yes

Are the conclusions drawn adequately supported by the results?

Yes

Are sufficient details of methods and analysis provided to allow replication by others?

Yes

Reviewer Expertise:

Antennas, Microwave, Electronic Circuits, Electromagnetics, Wireless Communication, underwater communication

We confirm that we have read this submission and believe that we have an appropriate level of expertise to confirm that it is of an acceptable scientific standard.

# Response

**Published:** 2024-09-03

**Authors:** SRIDHAR NAGISETTY

Dear Reviewer

 I would like to thank you for your valuable comments. I have amended the manuscript as per your comments and the corresponding point-to-point response is given below:

**Reviewer Comment-1:** The article is well written, and the results are relatively interesting, which I believe that the manuscript can provide some good insights for a future decision regarding 5G smart grid.

**Response**: Thank you for your valuable comment on our manuscript.

**Reviewer Comment-2:** However, as the class-J PA is well established, the authors should explain the novelty/contribution in the proposed class-J PA design

**Response**: Thank you, Prof., As per your suggestion, the main contribution/novelty of the proposed class-J PA is included as a **subsection** **before the conclusion section** in the manuscript.

**Reviewer Comment-3: **In Figure 6, authors are suggested to explain clearly how the phase shift and voltage boost are obtained by moving Alpha factor (α) from 0 to 1, in the relevant page?

**Response: **Thank you, Prof., As per your suggestion, the explanation for how the phase shift and voltage boost are obtained by moving the Alpha factor (α) from 0 to 1, is included **below Figure 6** in the relevant page.

**Reviewer Comment-4: **I would suggest the author to explain the reason for designing the matching networks of proposed PA by considering its optimum impedances corresponding to PAE rather than output Power.

**Response**: Thank you, Prof. As per your suggestion, I have explained the reason for designing the matching networks of the proposed class-J PA by considering its optimum impedances corresponding to PAE rather than output Power in the “ **Validation of optimum impedances obtained from load pull**” section.

**Reviewer Comment-5: **How is the second harmonic condition of class-J mode operation satisfied?

**Response: **Thank you, Prof., As per your suggestion, the explanation for how the second harmonic condition of class-J mode operation is satisfied is included **below Figure 15 **and before the **Results** section.

**Reviewer Comment-6: **Although the PAE and D.E of proposed PA are good its B.W is relatively low as per the comparison table, it is suggested to give some justification for obtained B.W of the proposed PA.

**Response: **Thank you, Prof., As per your suggestion, the justification for obtained BW of the proposed class-J PA is included in the discussion section **before table 4.**

**Reviewer Comment-7: **Table 4 gives the performance comparison of class -J PA. Can the authors report this class -J pa is relatively efficient than other PA types also.

**Response: **We report that the proposed class-J PA design of this work is not relatively efficient than other switching mode PAs. However, the proposed class-J PA has achieved high efficiency comparatively with the existing author’s class-J PA designs without sacrificing the linearity over the desired BW. This is included in the discussion section just **before Table 4**.
